# Acid is a potential interferent in fluorescent sensing of chemical warfare agent vapors

**DOI:** 10.1038/s42004-021-00482-6

**Published:** 2021-03-26

**Authors:** Shengqiang Fan, Genevieve H. Dennison, Nicholas FitzGerald, Paul L. Burn, Ian R. Gentle, Paul E. Shaw

**Affiliations:** 1 Centre for Organic Photonics & Electronics, School of Chemistry and Molecular Biosciences, The University of Queensland, Brisbane, QLD Australia; 2 Land Division, Defence Science & Technology Group, Fishermans Bend, VIC Australia

**Keywords:** Sensors, Fluorescence spectroscopy, Fluorescent probes, Chemical safety, Pollution remediation

## Abstract

A common feature of fluorescent sensing materials for detecting chemical warfare agents (CWAs) and simulants is the presence of nitrogen-based groups designed to nucleophilically displace a phosphorus atom substituent, with the reaction causing a measurable fluorescence change. However, such groups are also basic and so sensitive to acid. In this study we show it is critical to disentangle the response of a candidate sensing material to acid and CWA simulant. We report that pyridyl-containing sensing materials designed to react with a CWA gave a strong and rapid increase in fluorescence when exposed to Sarin, which is known to contain hydrofluoric acid. However, when tested against acid-free diethylchlorophosphate and di-*iso*-propylfluorophosphate, simulants typically used for evaluating novel G-series CWA sensors, there was no change in the fluorescence. In contrast, simulants that had been stored or tested under a standard laboratory conditions all led to strong changes in fluorescence, due to acid impurities. Thus the results provide strong evidence that care needs to be taken when interpreting the results of fluorescence-based solid-state sensing studies of G-series CWAs and their simulants. There are also implications for the application of these pyridyl-based fluorescence and other nucleophilic/basic sensing systems to real-world CWA detection.

## Abstract (web summary)

Chemical warfare agents and simulants are commonly detected with fluorescent sensing materials containing nitrogen-based groups, however these groups’ basicity can cause false positives in the presence of acids. Here, the authors disentangle the response of pyridyl-containing sensing materials to acid-containing and acid-free Sarin and simulants.

## Introduction

Highly toxic organophosphorus (OP) compounds have been developed as chemical warfare agents that target the nervous system^1^. Inhalation of the nerve agent or sorption through the skin or eyes leads to acetylcholinesterase (AChE) inhibition and an increase in acetylcholine^2^, which results in muscle overstimulation and potentially death. Nerve agents are conventionally divided into two main classes, the G- and V-series. Given the toxicity of nerve agents and reports of their use in both military and civilian settings, there is a drive to develop technologies that the military, first responders (national security), healthcare and environmental monitoring agencies can deploy in the field to confirm their presence. While methods such as gas chromatography-mass spectroscopy (GC-MS)^3^, enzyme-based biosensors^4–6^, chemiresistors^7–9^, and surface acoustic wave (SAW) sensors can be used for the detection of nerve agents^10,11^, they can suffer from a range of issues including being slow, nonselective, expensive, or simply too cumbersome for field use. Building on the success of fluorescence-based detection of explosives,^12–15^ there has been an increasing number of reports of using a change in a fluorescence signal for the sensing of nerve agents^16–18^. Given the electrophilic nature of the phosphorus atom in nerve agents, it is not surprising that the most commonly used strategy has been to develop a nucleophilic organic sensing compound that can react with the nerve agent^19–51^, although organometallic sensing materials have also been studied^52–55^. The design logic is based on the nucleophile of the sensing compound displacing one of the groups attached to the phosphorus atom, and generating a product whose fluorescence is different in either intensity or wavelength to that of the original sensing material. Nitrogen and oxygen-based nucleophiles have been most commonly used for organic sensing compounds designed to detect nerve agents^19–51^, with the pyridyl moiety (or its fused ring analogs quinoline and quinoxaline) featuring as the nucleophilic group in the majority of the sensing materials^19–30,42–46^. However, a key issue that needs to be addressed for nerve agent detection is selectivity, not just between different nerve agent classes^56^, but also from potential sources of false-positive responses. For example, nitrogen-containing nucleophiles are bases and so, in principle, simple acids could also cause a change in the fluorescence signal and hence act as interferents.

The role of acids as interferents is important, particularly for the G-series nerve agents that contain a fluorine-phosphorus bond (e.g., Sarin, Soman, and cyclosarin). The fluorine-phosphorus bond is susceptible to hydrolysis to form an organophosphoric acid and hydrofluoric acid^57^ and in fact, hydrofluoric acid is a known contaminant in as-prepared nerve agents^58^. Furthermore, given the toxicity of nerve agents and their restricted availability, the efficacy of new sensing materials is generally tested against simulants that have lower toxicity. The reported simulants used for Sarin, Soman, and cyclosarin detection are diethylchlorophosphate (DCP) and di-*iso*-propylfluorophosphate (DFP), with the former being the more widely used. Studies of the hydrolysis of DFP^59^ and DCP^60^ show that DFP reacts with water within a few hours, with the process accelerating when the measurements were done in glass containers^61,62^, while DCP reacts more quickly due to the weaker phosphorus-chlorine bond^60^.

When surveying the literature on the fluorescence-based detection of G-series nerve agents and simulants a number of observations can be made, particularly of the pyridyl-containing sensing materials that are the focus of this study. First, in many cases, the response of the sensing material to DCP and hydrochloric acid/hydrogen chloride (and indeed other acids) is the same. Second, the source and/or quality of the simulant is not described. Third, there is a significant number of publications that state that the product of the sensing process is the pyridinium hydrochloride salt that is formed via a phosphorylated intermediate. In most cases, no evidence is provided for the intermediate and it is assumed that hydrolysis occurs to give the salt. In the small number of cases where there is some apparent evidence, the measurements are undertaken in solution but without any indication of the kinetics of the reaction. Finally, given that solid-state reactions can be slower than those in solution there is an interesting question as to whether what is observed in solution is relevant for film-based detection, which is required for real time in field testing. That is, the solid-state response may be dominated by the acid impurity rather than the more complex multistep process of simulant diffusion into the film, phosphorylation, and hydrolysis.

**Fig. 1:**
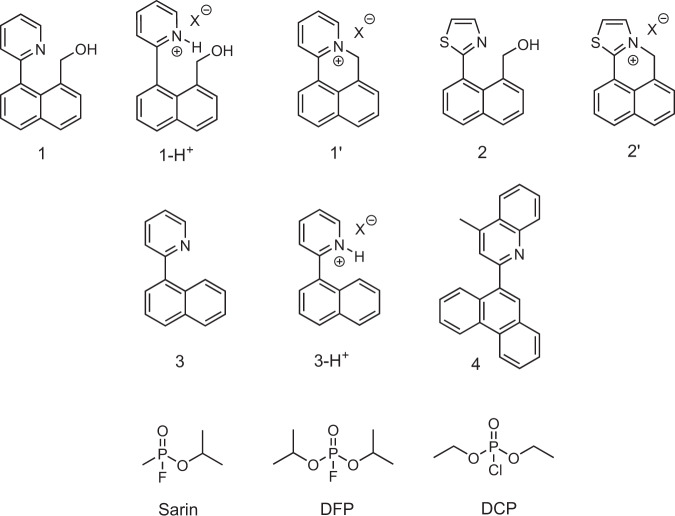
Structures of the sensing compounds, proposed products and analytes. Structures of compounds **1**, **2**, **3** and **4**, the protonated forms **1-H**^**+**^ and **3-H**^**+**^, the proposed cyclised products **1’** and **2’**, the analyte Sarin and its two simulants DFP and DCP. X is the counterion formed from the analyte vapor used.

Our current work was inspired by the need to develop a fundamental understanding of the role of acid as a potential interferent, particularly for sensing materials that contain the pyridyl moiety (or its fused ring analogs), which constitute around 50% of the reports at this time^19–30,42–46^. In the first part of the study, we explore a conceptually elegant substitution-cyclisation strategy for the detection of G-series CWAs. For example, it has been previously reported that 1-hydroxymethyl-8-(pyrid-2-yl)naphthalene **1** (Fig. 1) can be used to rapidly detect DCP and DFP^46^. The proposed mechanism involved the reaction of the primary alcohol to form a phosphate ester, followed by cyclisation to give pyridinium salt **1’** (Fig. 1). There have been a number of subsequent reports using this strategy^40–51^ although it has been noted that the pyridyl unit is susceptible to acid impurities. In investigating this strategy we replace the pyridyl moiety with a thiazolyl unit (**2** in Fig. 1) to compare the sensing efficacy and in so doing demonstrate the effect of acid impurities in the simulants on the detection process. The thiazolyl unit was chosen as the Ka of its conjugate acid is almost three orders of magnitude lower than pyridine (pKa = 2.5 versus 5.2) and, hence, should be less susceptible to acid interferents. We then expand the investigation to sensing materials that incorporate pyridyl groups more broadly (**3** and **4**, Fig. 1).

We show that if the Sarin or simulant is acid free there is no prompt response (change in the fluorescence) although there is a slow delayed response commensurate with the degradation of the simulant via hydrolysis under the measurement conditions. Our results are consistent with the view that the prompt fluorescence response of the sensing films upon Sarin or simulant vapor exposure is the result of protonation of the sensing material by acid impurities in the analyte, rather than the previously-proposed phosphorylation reaction pathways. Thus, it is important to carefully reconsider many of the reported sensors for the phosphonofluoridate G-series nerve agents and the effect of acid false positives.

## Results and discussion

### Purity and stability of the simulants

**Fig. 2:**
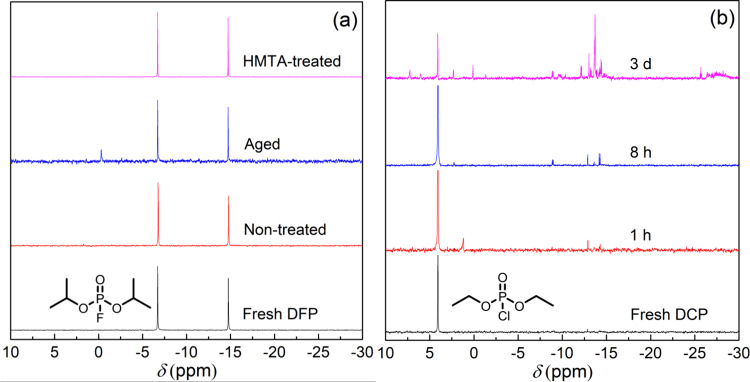
^31^P NMR spectra of DFP and DCP. **a** Fresh DFP, non-treated DFP (stored for 14 days), aged DFP (stored for 143 days) and HMTA-treated DFP (stored for 240 days); **b** Fresh DCP, and DCP stored in a capped glass vial for 1 h, 8 h, and 3 d without inert gas protection. The spectra were measured in dichloromethane-d_2_.

In the first part of this study, we investigated the purity and stability of DFP under different storage conditions. The DFP was synthesized following a reported procedure using di-*iso*-propylphosphite, cupric chloride, and cesium fluoride^63^, with the material purified by Kügelrohr (bulb-to-bulb) distillation (fresh DFP – experimental note: a tube filled with potassium carbonate and two cold traps were placed between the vacuum pump and Kügelrohr distillation apparatus to protect the pump). The synthesized DFP was sealed in a plastic bottle and stored at −20 °C for 14 days or 143 days (non-treated and aged, respectively), or stored over the acid scavenger, hexamethylenetetramine (HMTA – 20 mg HMTA was added to 200 μL DFP), for 240 days at −20 °C. The ^31^P NMR spectra (Fig. 2a) of the freshly prepared DFP had a doublet at δ = −10.71 ppm corresponding to the signal of the phosphorus atom with fluorine coupling (*J*_P-F_ = 972.8 Hz). No change in the spectrum was observed for the DFP stored over HTMA, even after 8 months. In contrast, the aged DFP stored at the same temperature was found to have a clear extra peak in the ^31^P NMR spectrum that corresponded to di-*iso*-propylphosphoric acid (δ = −0.31 ppm). It should be noted that given the sensitivity of ^31^P NMR it is possible that freshly prepared DFP also contained trace amounts of acid impurities. We found that acid impurities could be removed by treatment of the DFP with anhydrous potassium carbonate (10 wt%) for 30 min prior to an NMR measurement. However, DFP could not be stored over anhydrous potassium carbonate, with gel-like impurities forming on the surface of the particles during storage at −20 °C.

Based on the P-Cl and P-F bond strengths it would be expected that DCP would be more susceptible to hydrolysis than DFP. A previous ^31^P NMR study on the hydrolysis of DCP showed that when water was in excess diethyl phosphoric acid was formed, but when the DCP was used in large excess then multiple products corresponding to the phosphate monomer (^31^P NMR −5.00 ∼ 5.00 ppm), pyrophosphate dimer (^31^P NMR −20.00 ∼ −5.00 ppm) and poly(phosphate) trimer (^31^P NMR −30.00 ∼ −25.00 ppm) were observed^60^. DCP is often used under ambient conditions. We, therefore, investigated the stability of DCP to ambient moisture by placing 200 μL of freshly distilled DCP stored in a glass vial (2 mL) that had been capped under normal laboratory environmental conditions, that is room temperature with the relative humidity being around 50–60%. The ^31^P NMR of the DCP was measured over intervals of time with the results shown in Fig. 2b. Freshly distilled DCP had a peak at 4.06 ppm, but after an hour, extra signals in the ^31^P NMR were observed. These signals continued to grow over time and were consistent with the previously reported hydrolyzed products^60^. In this experiment, DCP absorbs water from the atmosphere and so DCP is always present in large excess relative to water. The fact that phosphorous impurities form means that hydrochloric acid will also be present in the DCP.

These results demonstrate the importance of knowing the storage history of DFP and DCP when testing new fluorescence-based sensing materials that are capable of acting as a Brønsted or Lewis base. Failure to do so could lead to acid impurities giving a false positive response to a simulant, even when the acid is present at very low levels. It should be noted that the impurity and storage of DFP can also be influenced by the synthetic method. For example, DFP synthesized with di-*iso*-propyl phosphite, potassium fluoride and 1,3-dichloro-5,5-dimethylhydantoin has been reported to show better storage stability than commercial DFP^61^.

### Thin-film sensing of Sarin, DFP, and DCP vapors

**Fig. 3:**
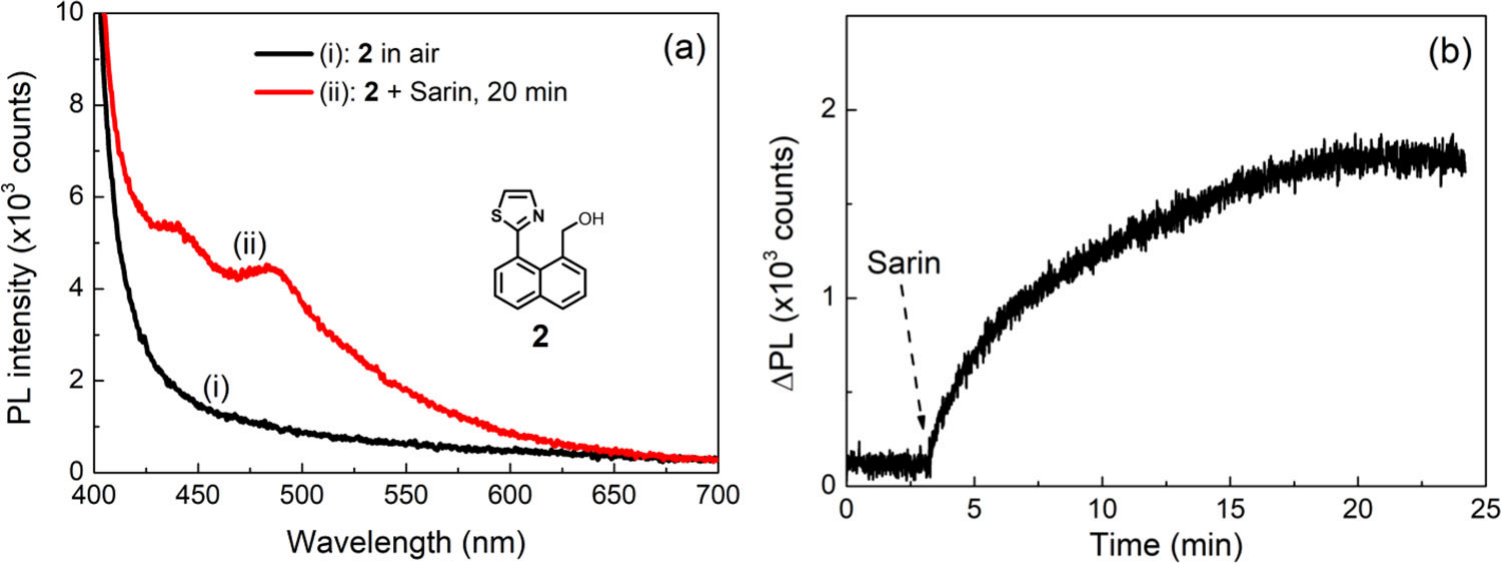
PL response of sensing compound 2 to Sarin. PL spectra (**a**) and time-dependence (**b**) of solid-state films with **2** in cellulose acetate before and after the introduction and continuous exposure to the vapor of a stored Sarin sample. A 365 nm LED was used to excite the film. The PL spectra were recorded on an OceanOptics Flame spectrometer and were not corrected. The PL signal in the absence of Sarin in **a** is due to the tail of the excitation. For the PL time-dependence, the intensity of the emission peak at 484 nm was recorded and the data has been corrected for the scattered excitation.

Following the method reported for vapor detection using **1** we prepared thin films containing 10 wt% of **2** (see Supplementary Methods for the synthetic route) in cellulose acetate for the detection of Sarin vapor. The as-prepared film was found to be non-emissive when excited at 365 nm (note that **2** does not absorb at this wavelength) (Fig. 3a, curve i) but when exposed to the vapor of Sarin that had been stored, a new emission peak at 484 nm was observed (Fig. 3a, curve ii), with the photoluminescence (PL) intensity showing a gradual increase over time (Fig. 3b).

**Fig. 4:**
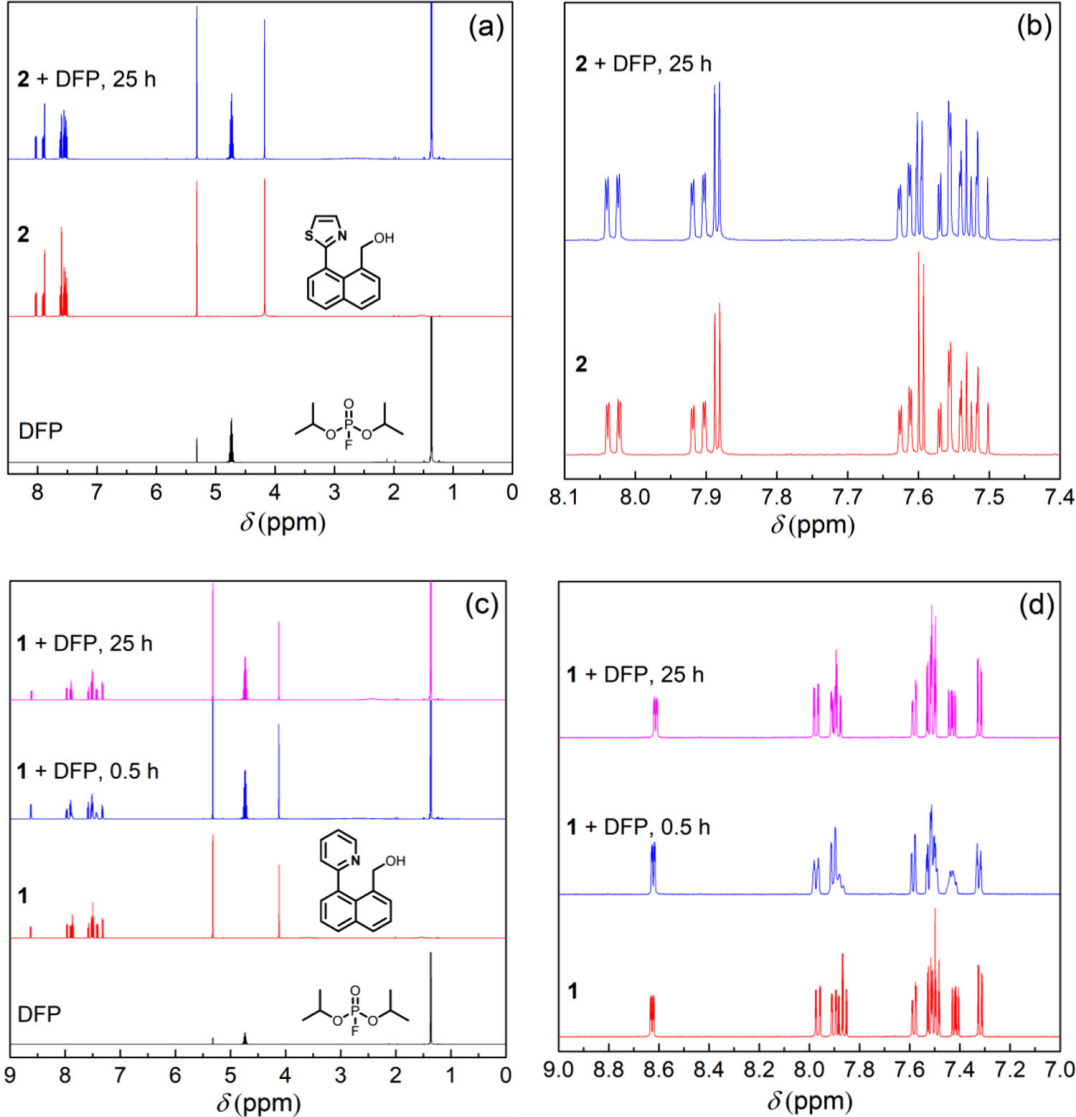
^1^H NMR spectra of the sensing compounds upon addition of DFP. Spectra of **2** (**a** full spectrum; **b** aromatic region) and **1** (**c** full spectrum; **d** aromatic region) with addition of DFP (1 eq) in dichloromethane-d_2_. All experiments were carried out at room temperature.

Based on the solution-based measurements reported for **1** in the presence of DCP and DFP, the fluorescence turn-on observed for the film containing **2** would be consistent with it undergoing a rapid nucleophilic substitution-cyclisation process akin to **1** being converted to **1’**. To confirm this was indeed the case, we followed the reaction of **2** with one equivalent of freshly prepared DFP (acid-free) using solution ^1^H NMR (Fig. 4a: full spectrum; Fig. 4b: aromatic region). Given the rapidity of the film response to Sarin we were surprised to find that even after 25 h no new ^1^H NMR peaks corresponding to the cyclised product were observed. Thus, while there appeared to be a rapid response to Sarin vapor in the film, in solution the reaction with the simulant was essentially non-existent. The results are counter to what would normally be expected, that is, solution-based chemical transformations are often faster than those in the solid-state, particularly when there is a significant requirement for structural rearrangement.

It was therefore deemed critical to determine why we observed an increase in the PL with Sarin and no chemical transformation for DFP in solution. The relative electrophilicities of DFP and Sarin could be one source of the response difference and to confirm whether this was the case we compared the response of a solution of **2** with that of **1**, which has been reported to react with DFP both in solution and film^46^. As stated earlier the Ka of the conjugate acid of thiazole is three orders of magnitude lower than that of pyridine and hence the nitrogen of **2** is less nucleophilic than that of **1**. Furthermore, the cyclisation product from **2** would be expected to have a higher ring strain, which could also explain its lack of formation as compared to **1**. However, it should be noted that the ^1^H NMR experiment appeared to show that the first step, the reaction with the benzylalcohol of **2**, had also not occurred. We, therefore, carried out the same ^1^H NMR experiment with **1** as the sensing compound, with the results shown in Fig. 4c (Fig. 4d: aromatic region). Again, no new peaks corresponding to the cyclised product (in this case **1’** with X = F) were observed after 25 h, which is inconsistent with the reported rapid change in fluorescence both in solution and film^46^.

**Fig. 5:**
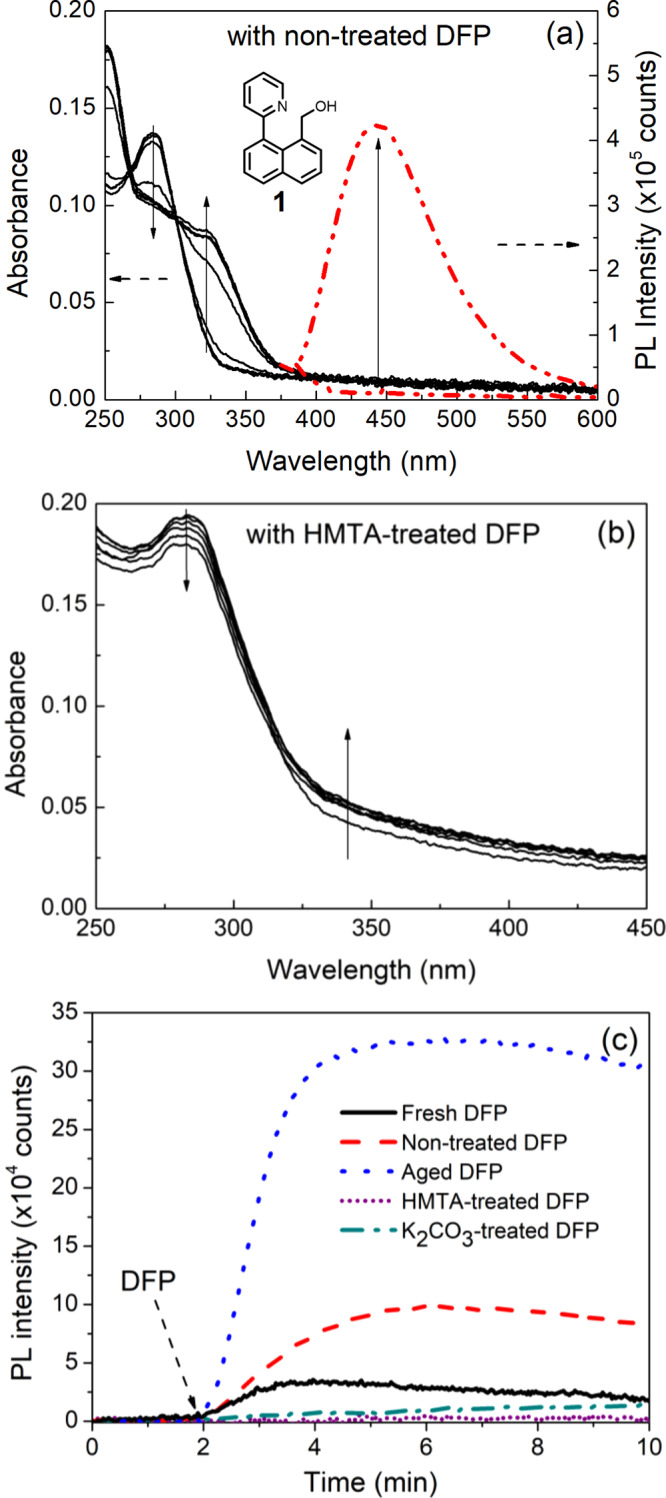
Sensing results of compound 1 upon exposure to differently treated DFP. **a** UV–vis absorption and PL spectra (excitation wavelength = 365 nm) of 10 wt% **1**:cellulose acetate films upon exposure to non-treated DFP (0–4 min); **b** UV–vis absorption spectra of the films upon exposure to HMTA-treated DFP (0–10 min); **c** Representative PL kinetics curves for the sensing films against various DFP sources. The PL spectra in **a** were recorded using an FS5 spectrometer and the PL kinetics in **c** were recorded using an OceanOptics Flame spectrometer using the setup described in Supplementary Fig. S2a.

These ^1^H NMR experiments clearly showed that neither **1** or **2** undergo an appreciable chemical transformation with DFP and hence the change in fluorescence shown in Fig. 3 must arise from a different process. As described earlier, both DFP and DCP are known to hydrolyze to give hydrofluoric and hydrochloric acid, respectively, and the presence of acid could potentially be the source of the observed change in fluorescence of the films when exposed to Sarin. We, therefore, exposed **1**:cellulose acetate blend films to vapors of DFP that had been stored and/or treated under different conditions. Compound **1** was chosen to enable direct comparison with the previously reported results^46^. When the 10 wt% **1**:cellulose blend film was exposed to non-treated DFP (stored at −20 °C for 14 days) the absorption spectrum was red shifted with a new peak at 322 nm and the onset shifted from ~350 nm to ~400 nm (Fig. 5a). In addition, fluorescence turn-on at 444 nm was observed (excitation wavelength = 365 nm) (Fig. 5a). It should be noted that a similar response was achieved when **1** was blended with cellulose acetate at a 1 wt% concentration (Supplementary Fig. S1). When the vapor from freshly prepared DFP was used there was a much smaller increase in the fluorescence than that of the aged-DFP (143 days of storage) (Fig. 5c). The acid-free HMTA-treated DFP showed no observable changes in either the absorption (Fig. 5b) or PL spectra (Fig. 5c). The difference in magnitude of these responses was consistent with acid being present in the DFP based on the hydrolysis products observed in the ^31^P NMR spectra (Fig. 2). Thus, combining the results from the ^1^H NMR and film PL measurements strongly suggests that neither **1** and **2** are responding to Sarin or the simulant but rather the acids formed from their hydrolysis.

**Fig. 6:**
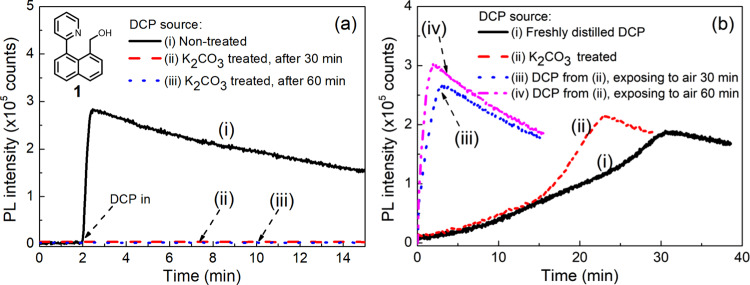
Sensing results of compound 1 upon exposure to differently treated DCP. Representative PL kinetics curves for 10 wt% **1**:cellulose acetate sensing films under (**a**) a nitrogen flow containing non-treated DCP or potassium carbonate-treated DCP (30 or 60 minutes), and (**b**) an air atmosphere with freshly distilled DCP (curve i), potassium carbonate-treated DCP (curve ii), and potassium carbonate-treated DCP left in air for 30 (curve iii) and 60 min (curve iv). The PL kinetics were recorded using an OceanOptics Flame spectrometer.

We also carried out a similar series of experiments with DCP, which as described earlier is known to hydrolyze faster than DFP. The DCP was purified by distillation and stored in a nitrogen-filled glovebox. We first performed the sensing measurement under an inert atmosphere. The DCP vapor was generated by bubbling nitrogen through DCP liquid (Supplementary Fig. S2b). When films of **1**:cellulose blends were exposed to the glovebox-stored DCP vapor a rapid fluorescence turn-on (within 30 s) was observed, as shown in Fig. 6a. Even though we took care to store the DCP under anhydrous conditions, we were concerned that the rapid increase in fluorescence could be due to acid impurities formed during storage or transfer. To test this, we added anhydrous potassium carbonate to the DCP, which was then stirred for 30 min before use. Repeating the experiment with the potassium carbonate-treated DCP under the same conditions as before led to no observable change in fluorescence (Fig. 6a), confirming that removing the acid impurities from DCP is critical. An important side observation from this experiment is that it shows that the reaction between DCP and the hydroxyl groups of the cellulose acetate is slow relative to the sensing process in the solid-state. In a final experiment, we investigated the ability of the **1**:cellulose films to sense DCP in air. Using a similar method to that of the DFP experiments, the DCP was placed at the bottom of the optical sensing chamber in air (Supplementary Figure S2a), and the change in fluorescence measured over time. As shown in Fig. 6b, when carrying out the measurements immediately using freshly distilled DCP (curve i) or DCP stored over potassium carbonate (curve ii) a fluorescence turn-on response was observed, although the change was relatively slow (curve i). If the DCP was left in the chamber in air for 30 min to an hour before the measurement, then the change in fluorescence was rapid (curves iii and iv, respectively). These results indicate that DCP rapidly reacts with moisture in the air and that the results from measurements undertaken under such conditions should be treated with caution and potentially re-evaluated.

**Fig. 7:**
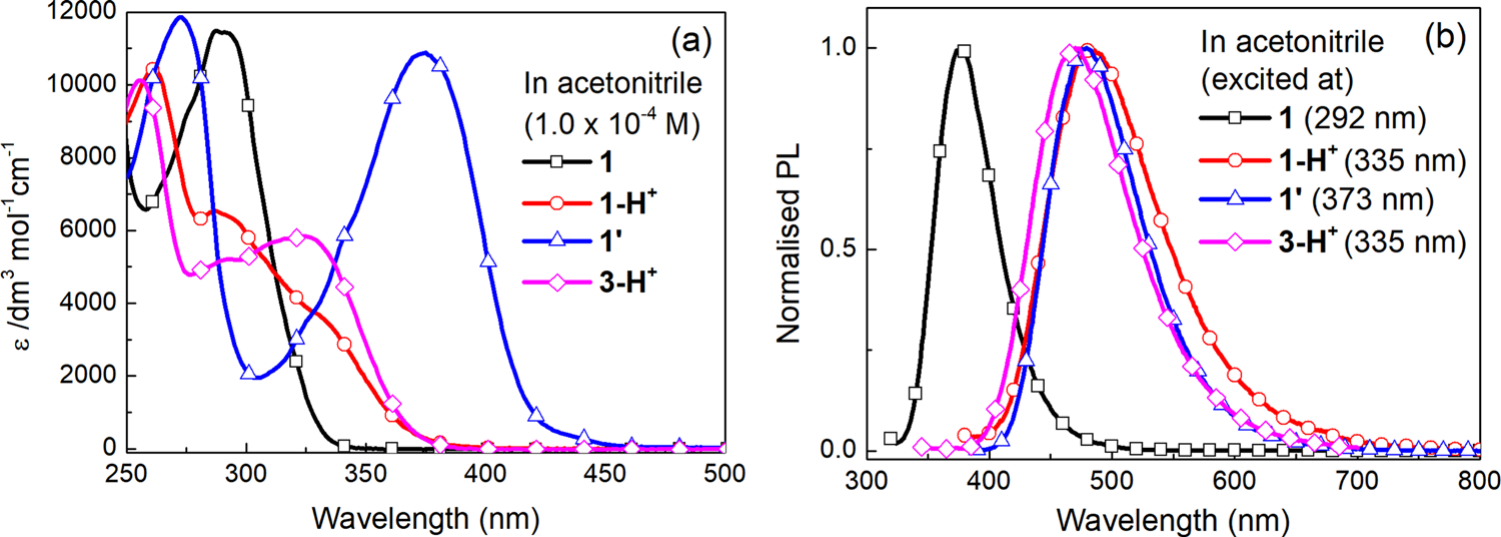
The optical properties of 1, 1-H^+^, 1’, and 3-H^+^ in acetonitrile. **a** UV–vis and **b** PL spectra.

In a further set of experiments to confirm that the change in fluorescence observed for **1**, and by analogy **2**, was due to acid and not the simulant, we prepared the protonated product **1-H**^**+**^ (X = Cl) and the fully cyclised compound **1’** (X = Br) (see Supplementary Methods for the synthetic procedures and Supplementary Figs. S3 and S4 for ^1^H NMR analysis of the structures). We also prepared **3**^64^ that lacked the alcohol group required for the initial nucleophilic substitution step with the simulant and its protonated form **3-H**^**+**^ (X = Cl). Figure 7 shows the absorption (Fig. 7a) and PL (Fig. 7b) spectra of the compounds in acetonitrile. In Fig. 7a it can be clearly seen that the onset of the absorption spectrum of **1-H**^**+**^ (X = Cl) is red-shifted compared to **1** and similar to that of **3-H**^**+**^ (X = Cl). However, the long-wavelength components of the absorption spectra of the protonated compounds are significantly blue-shifted when compared with the cyclised compound **1’** (X = Br) whose absorption peak was at 374 nm. It is interesting to note that the PL spectra of **1-H**^**+**^ (X = Cl), **3-H**^**+**^ (X = Cl), and **1’** (X = Br) are at similar wavelengths but red-shifted compared to **1**. It should also be noted that the protonated and cyclised compounds showed a solvochromatic effect (see Supplementary Fig. S5), which needs to be considered when comparing results with published reports and solid-state measurements.

**Fig. 8:**
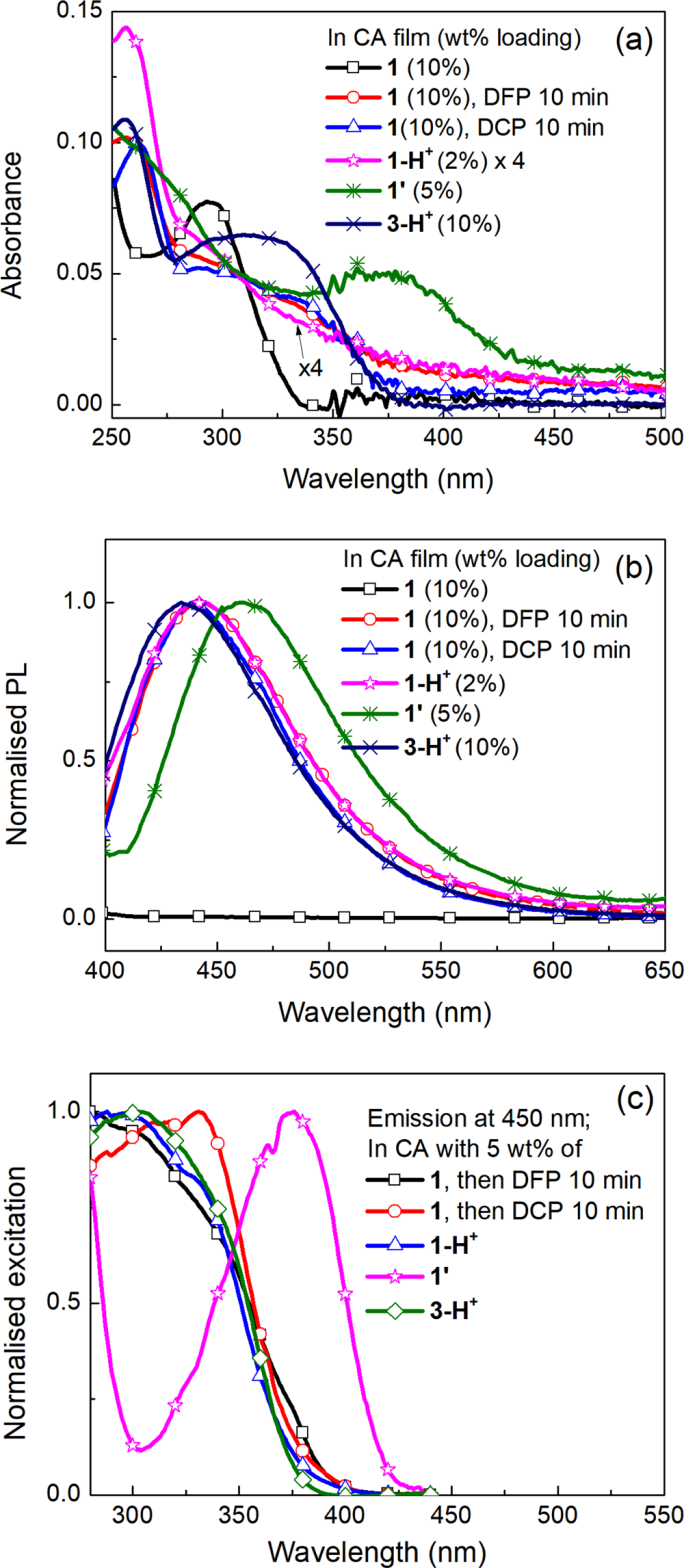
The optical properties of 1, 1-H^+^, 1’, and 3-H^+^:cellulose acetate films compared to 1:cellulose acetate films upon exposure to aged DFP or non-treated DCP vapor. **a** UV–vis, **b** emission (excitation wavelength = 365 nm), and **c** excitation spectra measured for an emission wavelength of 450 nm.

The optical properties of the compounds in cellulose acetate films were then investigated and compared with the films of **1** exposed to aged DFP or non-treated DCP (Fig. 8). The concentration of **1** or **3-H**^**+**^ (X = Cl) was 10 wt% (in a similar manner to the sensing measurements shown in Fig. 6). However, due to solubility limitations and the need to have optically clear films for the UV–vis absorption measurements, the concentrations of **1-H**^**+**^ (X = Cl) and **1’** (X = Br) were 2 and 5 wt%, respectively. Figure 8a shows that the absorption maximum of **1’** (X = Br) was at the longest wavelength in a similar manner to the solution measurements. Importantly, the absorption spectra of the films of **1** exposed to aged DFP and non-treated DCP were similar to that of the **1-H**^**+**^ (X = Cl):cellulose acetate and **3-H**^**+**^ (X = Cl):cellulose acetate films but significantly different to the **1’**:cellulose acetate film. Likewise, the PL spectra (Fig. 8b) of the **1**:cellulose acetate films exposed to aged DFP or non-treated DCP were similar to that of the **1-H**^**+**^ (X = Cl):cellulose acetate film, with the peak blue-shifted compared to the cyclized **1’**:cellulose acetate film. The excitation spectra (Fig. 8c) provide further spectroscopic evidence that the fluorescence turn on is due to protonation and not cyclisation. The excitation spectra were measured at an emission wavelength of 450 nm and it can be clearly seen that the spectra of the **1**:cellulose acetate films exposed to aged DFP or non-treated DCP are the same as those of the **1-H**^**+**^:cellulose acetate films and different to the spectrum of the **1’**:cellulose acetate film.

When these photophysical results are taken in conjunction with the NMR data shown in Fig. 4 and the sensing measurements shown in Figs. 5c and 6a where there was no PL response for **1**:cellulose acetate films exposed to potassium carbonate or hexamethylenetetramine treated DFP or potassium carbonate-treated DCP the logical conclusion is that protonation due to acid impurities in the DFP and DCP is the cause of the rapid change in the fluorescence.

**Fig. 9:**
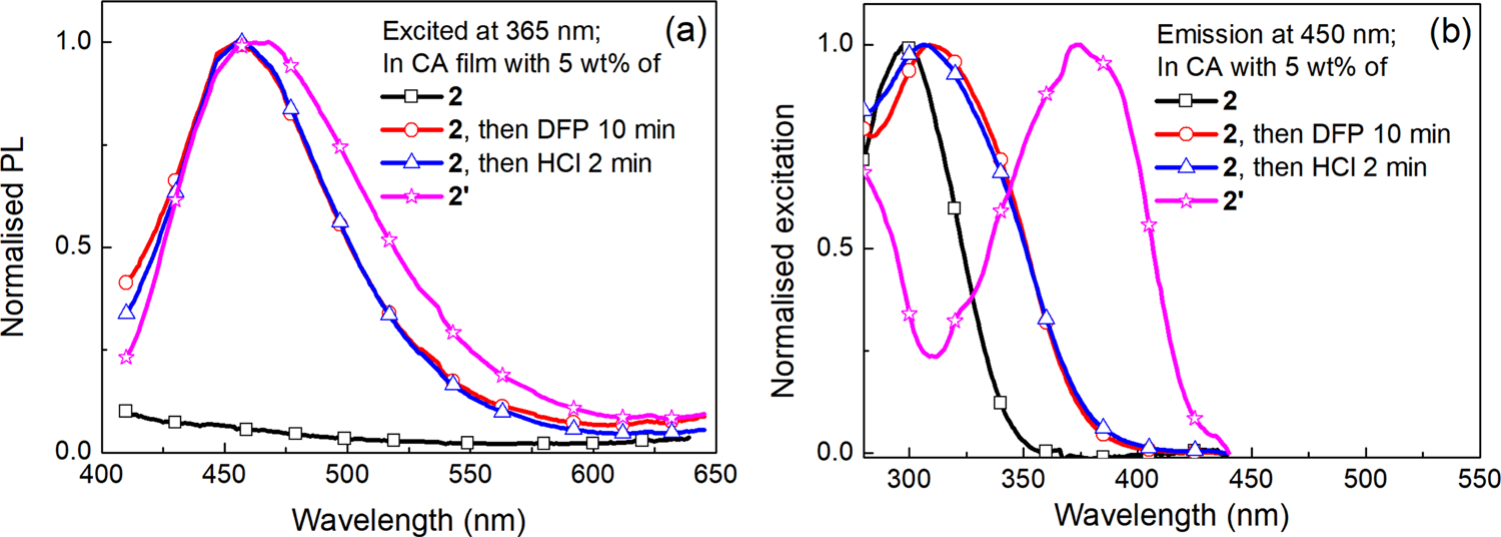
The optical properties of 2 and 2’:cellulose acetate films compared to 2:cellulose acetate films upon exposure to aged DFP or hydrogen chloride vapor. **a** PL emission and **b** excitation spectra. PL spectra were recorded using an FS5 spectrometer. DFP and HCl vapor were generated using the setup described in Supplementary Fig. S2a.

We also prepared the cyclised compound **2’** (X = Br) from **2** to confirm the difference in the photophysical properties we observed between **1** and **1’**. It should be noted that the low pKa (2.5) of the conjugate acid of thiazole meant we could not isolate protonated **2**. The emission and excitation spectra of films of **2** (before and after treatment with aged DFP or hydrogen chloride) and **2’** blended with cellulose acetate are shown in Fig. 9. The PL spectrum of the film containing **2’** was red shifted compared to those of the **2**:cellulose acetate films when exposed to aged DFP and hydrogen chloride (Fig. 9a). However, the excitation spectrum provides additional clear evidence that the increase in the fluorescence is due to protonation of **2** and not cyclisation to form **2’** (Fig. 9b). It can be clearly seen that the excitation spectrum at an emission wavelength of 450 nm of **2’** (X = Br) is significantly red-shifted compared to the films that had been treated with the aged DFP and hydrogen chloride, which were essentially the same. That is, the PL shown in Fig. 9a arises from the protonation of **2**.

**Fig. 10:**
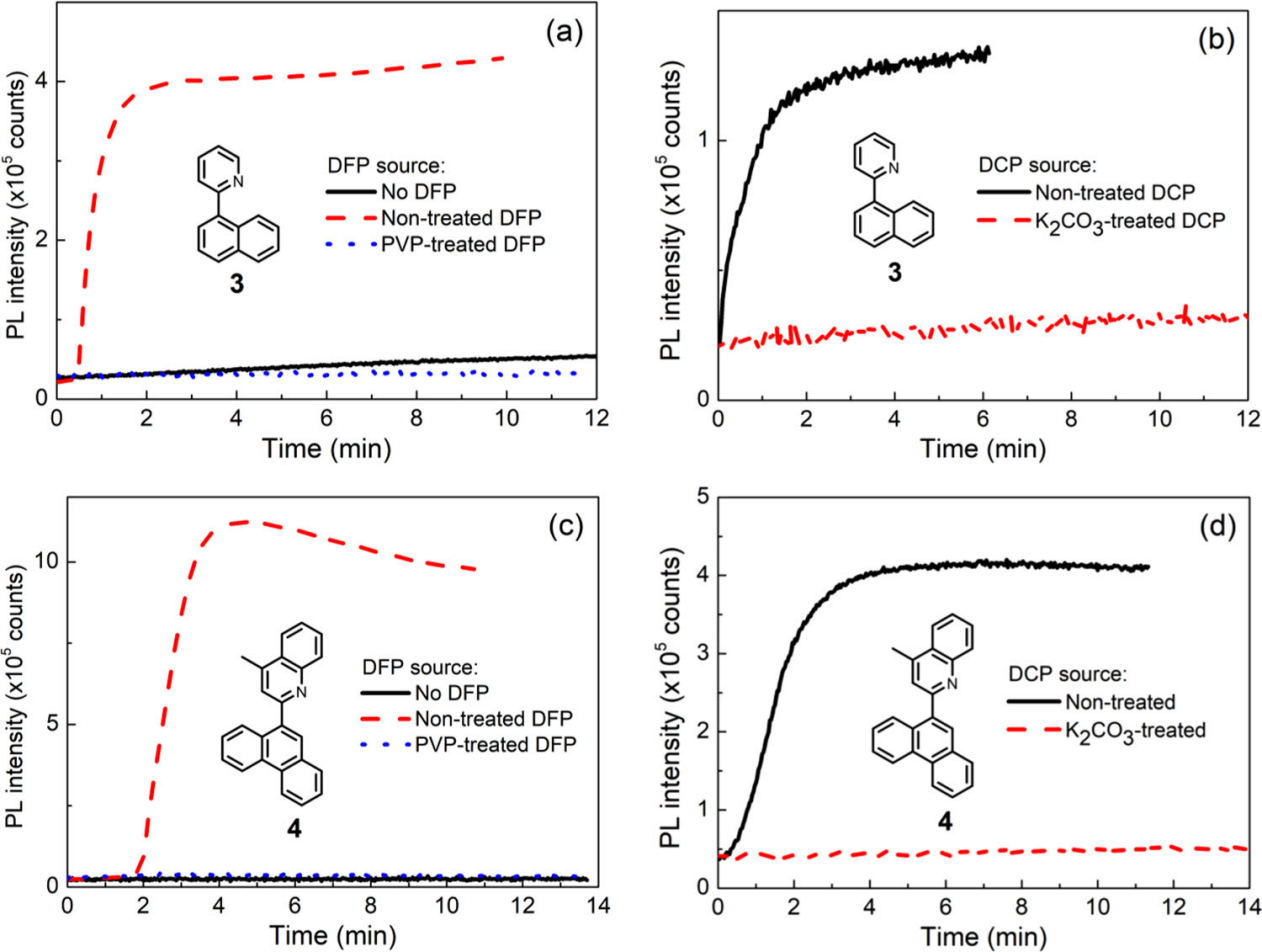
Sensing results of compounds 3 and 4 upon exposure to differently treated DFP and DCP. Representative PL kinetics curves for (**a**, **b**) 10 wt% **3**:cellulose acetate sensing films and (**c**, **d**) 10 wt% **4**:cellulose acetate sensing films exposed to acid free and impure DFP and DCP sources. The set-up shown in Supplementary Fig. S2a was used for measurements with DFP with that of Supplementary Fig. S2b used for DCP. Non-treated DFP was stored at −20 °C for 3 months; PVP-treated DFP was prepared by adding 20 mg of poly(4-vinylpyridine) (PVP) to 200 μL of DFP; Non-treated DCP was stored in a glovebox; potassium carbonate-treated DCP was prepared by adding 200 mg of potassium carbonate to 1 mL of DCP. The PL kinetics were recorded using an OceanOptics Flame spectrometer.

There are a significant number of sensing materials that contain the pyridyl moiety (and fused heteroaromatic rings such as quinoline and quinoxaline) that do not require the cyclisation step. We, therefore, explored **3** and **4**^65^ for the solid-state detection of Sarin and/or DFP and DCP vapor that either contained acid or were acid-free with the results shown in Fig. 10. It can be seen that both **3** and **4**:cellulose acetate blend sensing films showed a sharp fluorescence turn-on response when they were exposed to either non-treated DFP or DCP (Fig. 10a: **3** with DFP; Fig. 10b: **3** with DCP; Fig. 10c: **4** with DFP; Fig. 10d: **4** with DCP). Comparison of the response of the **4** containing films to DCP or hydogren chloride shows that the PL spectra of both films were identical (Supplementary Fig. S6). However, when DFP and DCP were treated with poly(4-vinylpyridine) (PVP) and anhydrous potassium carbonate, respectively, no response (Fig. 10a, c with DFP) or a very slow (Fig. 10b, d with DCP) change in the fluorescence was observed. These results are in agreement with the fluorescence response from compound **1** (Fig. 5c with DFP and Fig. 6a with DCP) and are evidence that the observed sharp fluorescence turn-on response with non-treated DFP and DCP is due to the acid impurities. Thus it is reasonable to conclude that published sensing materials containing pyridyl-like moieties detect the acid impurities rather than DFP or DCP themselves. Interestingly, nearly 20 years ago a ^31^P NMR study showed that the rate of reaction between pyridine and diethyl chorophosphate was slow with the equilibrium strongly to the left^66^. Furthermore, they found changes in the ^31^P NMR spectra occurred more rapidly in the presence of water. Thus, while it may be possible for a phosphoropyridinium intermediate to form in solution it is unlikely to be responsible for the rapid observable fluorescence changes in the detection of the vapors by sensing films. That is, the lack of response when **3** or **4** were blended with hydroxyl-containing cellulose acetate and exposed to base-treated (acid free) DFP or DCP was because the phosphoropyridinium intermediate and its subsequent hydrolysis by adventitious water simply does not occur on the time scale of the measurement.

**Fig. 11:**
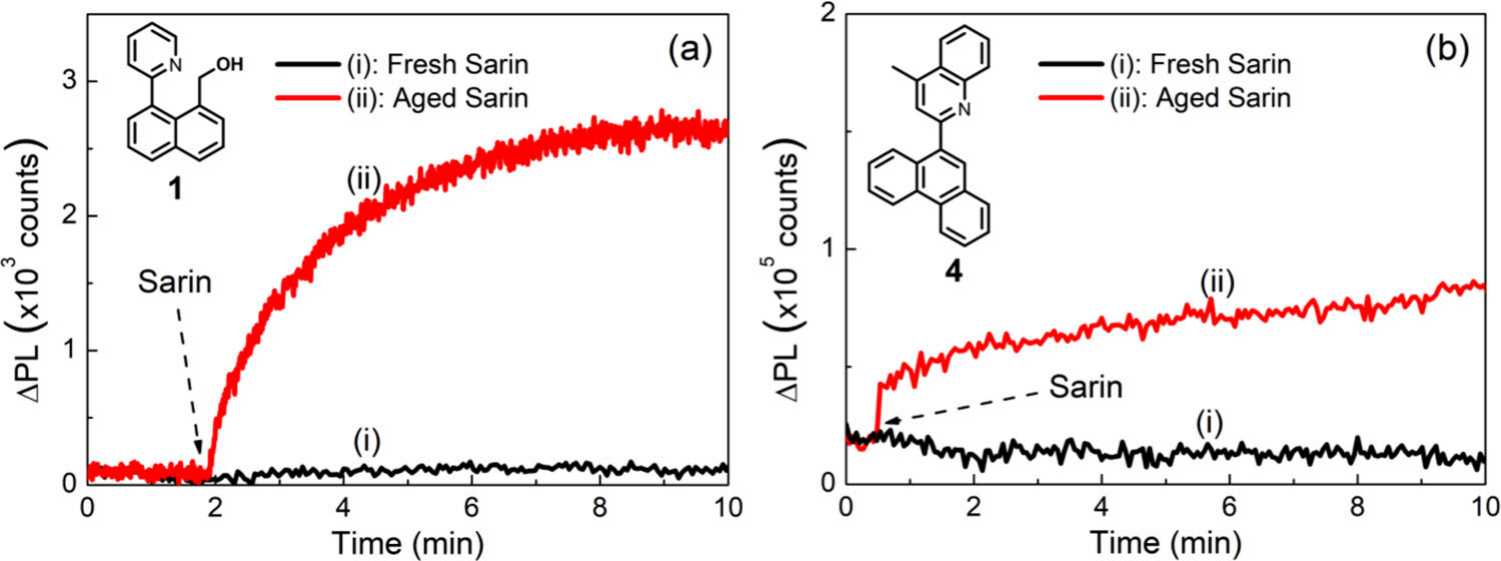
Sensing results of films of 1 and 4 upon exposure to freshly prepared and aged Sarin. Representative PL kinetics curves for (**a**) 10 wt% **1**:cellulose acetate and (**b**) 10 wt% **4**:cellulose acetate sensing films exposed to vapors of aged (being stored for more than 6 months) and fresh (prepared and distilled twice less than one week before use) Sarin using the Supplementary Fig. S2a setup. The PL kinetics were recorded using an OceanOptics Flame spectrometer.

Finally, to confirm that what we observed for the pyridyl-containing compounds with or without hydroxyl group, was not an artifact of the CWA simulants (DFP and DCP) we also tested films containing **1** and **4** against Sarin with different storage histories. The Sarin used was either aged (being stored for more than 6 months) or freshly synthesized (less than one week before use). As shown in Fig. 11, a fluorescence turn-on response was observed with the aged Sarin while there was no response for both compounds **1** and **4** when freshly prepared Sarin was used. Thus, these results further confirm that phosphorylation of the pyridyl-containing compounds is not the major mechanism for fluorescence detection of the G-series CWAs and their simulants.

## Conclusion

Drawing these results together, we have investigated sensing compounds that were designed with functionality to undergo a substitution or substitution-cyclisation pathway for the detection of phosphonofluoridate G-series nerve agents and simulants. The results show that the fast fluorescence response of the sensing thin films upon Sarin, DFP or DCP vapor exposure are the result of simple protonation of the sensing material by acid in the analyte, rather than the previously-proposed phosphorylation and/or subsequent cyclisation. Indeed, in solution the sensing materials can detect acid at a concentration as low as 0.4 μM (Supplementary Fig. S7). Thus, these findings have significant implications for many of the developed sensing materials which have nucleophilic groups that are also basic and hence it is important to carefully reconsider the effect of acid false positives. Indeed it is of concern that the fluorescence responses of many of the reported sensors for the phosphonofluoridate G-series nerve agents may simply be caused by the presence of acid impurities in the simulant, particularly for solid-state detection of CWA vapors. We believe that the findings described here will stimulate the ongoing search for truly selective chemosensors for nerve agents.

## Methods

### Film preparation

The sensing compound was dissolved in ethanol (1–10 mg/mL). Cellulose acetate (Sigma-Aldrich, average M_n_ ~30,000 by GPC, 39.8 wt% acetyl) was dissolved in acetone (20 mg/mL). Then 200 μL of sensing compound in ethanol was mixed with 900 μL of cellulose acetate in acetone and the resulting mixture was stirred at room temperature for 10 min. Thin films were prepared on fused silica substrates by spin-coating at 2000 rpm for 60 s to give a thickness of ≈220 nm.

### Sensing measurements

*Caution*: Dialkylfluorophosphates are highly toxic compounds. They should only be synthesized, purified, used and disposed of after rigorous risk assessment. All processes should only be undertaken by trained persons using appropriate personal protection equipment. *Disclaimer*: Work with Schedule 1 CWAs should only be performed at OPCW declared facilities. The sensing film samples on fused silica substrates were mounted in a closed sample chamber which was connected via optical fibers to an LED light source (365 nm, OceanOptics) and a spectrometer (Flame, OceanOptics). The sample chamber featured three optical windows to allow for excitation of the films and subsequent detection of the film PL at right angles to the excitation. For experiments conducted in air (see Supplementary Fig. S2a for the experimental setup), 2 μL of DFP, DCP or hydrochloric acid (16%) was placed on the surface of a Teflon lid, which was placed at the bottom of the chamber or a pipette droplet of Sarin was added directly into the bottom of the chamber. Evaporation at 20–22 °C gave the analyte vapor. Film PL spectra before and after exposure to the analyte were recorded using an OceanOptics Flame spectrometer whose spectral response was not corrected or an FS5 spectrometer whose spectral response was corrected. PL kinetics at the emissive peak were recorded on the Flame spectrometer through OceanView software (OceanOptics). Due to the fast hydrolysis of DCP with moisture, sensing experiments with DCP were also carried out under a nitrogen flow (see Supplementary Fig. S2b for the experimental setup). Specifically, a mixture of DCP (1 mL) and anhydrous potassium carbonate (200 mg) was stirred in a 10-mL Schlenk tube at room temperature under nitrogen for 30 min. Then nitrogen (100 mL/min) was bubbled through the liquid DCP to generate the vapor, which was then diluted using a second nitrogen flow (200 mL/min), before being introduced into the optical chamber for the sensing measurement. The nitrogen stream was then passed through a scrubbing solution (20 wt% sodium hydroxide in water) to break down the excess simulant.

## Supplementary information

Supplementary Information

## Data Availability

The authors declare that data and experimental methods supporting the findings of this study are available in the Supplementary file. Unrestricted data is available from the authors upon reasonable request.
