# Supplementary material for: Acid is a potential interferent in fluorescent sensing of chemical warfare agent vapors
Source: Commun Chem. 2021 Mar 26;4:45. doi: 10.1038/s42004-021-00482-6 (PMC9814523; doi:10.1038/s42004-021-00482-6)
Supplement: Supplementary file 1 — Supplementary Information [file 42004_2021_482_MOESM1_ESM.pdf]

Acid is a potential interferent in fluorescent sensing of chemical warfare agent vapors

Shengqiang Fan[1], Genevieve H. Dennison[2], Nicholas FitzGerald[2], Paul L. Burn[1✉], Ian R. Gentle[1] & Paul E. Shaw[1✉]

[1]Centre for Organic Photonics & Electronics, School of Chemistry and Molecular Biosciences, The University of Queensland, Brisbane, QLD 4072, Australia. [2]Land Division, Defence Science & Technology Group, Fishermans Bend, Vic 3207, Australia. ✉email: p.burn2@uq.edu.au; p.shaw3@uq.edu.au

Supplementary Methods

Materials synthesis: All reagents were purchased from commercial sources and were used as received unless otherwise stated. Solvents for chromatography were distilled prior to use. Column chromatography was performed using Davisil LC60A 40–63 micron silica gel. ^{1}H and ^{13}C NMR were performed using Bruker Avance 500 MHz spectrometers in deuterated dichloromethane referenced to 5.32 ppm for ^{1}H and 53.8 ppm for ^{13}C; in deuterated methanol referenced to 3.30 ppm for ^{1}H and 49.0 ppm for ^{13}C; or in deuterated dimethyl sulfoxide referenced to 2.50 ppm for ^{1}H and 39.5 ppm for ^{13}C. Naphthalenyl-H = naphthalenyl H; Thiazole-H = thiazole H; Pyridyl-H = pyridyl H; Pyridinium-H = pyridinium H. Coupling constants are given to the nearest 0.5 Hz. UV-visible spectrophotometry was performed using either a Cary 5000 UV-Vis spectrophotometer or an OceanOptics Flame spectrometer. Solution measurements were carried using dichloromethane, acetonitrile or ethanol as solvent. Film measurements had the material deposited on quartz substrates. Absorbance shoulders are denoted as sh. Solution and film photoluminescence spectra and intensity were recorded using either an FS5 spectrofluorometer or an OceanOptics Flame spectrometer. FT-IR spectroscopy was performed on solid samples using a Perkin-Elmer Spectrum 100 FT-IR spectrometer with an ATR attachment. Melting points (MPs) were measured in a glass capillary on a Büchi B-545 melting point apparatus and are uncorrected. Microanalyses were performed using a Carlo Erba NA 1500 Elemental Analyzer. High resolution electrospray ionisation (HRMS) accurate mass measurements were recorded in positive mode on a Bruker MicroTOF-Q (quadrupole-time of flight) instrument with a Bruker ESI source. Thermal transitions were determined using a Perkin-Elmer Diamond Differential Scanning Calorimeter. Thermal gravimetric analysis was undertaken using a Perkin-Elmer STA 6000 Simultaneous Thermal Analyzer. Thermal decomposition temperatures ($T_{5\%}$) are reported as the temperature corresponding to a 5% mass loss.

The synthetic pathway to **2** is shown in Scheme S1 with **1** being prepared following the literature procedure[1]. In the first step commerical 2-(tri-*n*-butylstannyl)thiazole **5** was reacted with *tert*-butyl[(8-iodonaphthalen-1-yl)methoxy]dimethylsilane **6**[2] under Stille conditions to give **7** in a 98% yield. The silyl protecting group was then removed by treating **7** with aqueous acid to give **2** in an isolated yield of 66%. The protonated products **1-H**$^+$ (X = Cl) and **3-H**$^+$ (X = Cl) were synthesized by treatment of **1** or **3** in diethyl ether with anhydrous hydrogen chloride[3]. The cyclic compounds **1'** (X = Br) and **2'** (X = Br) was synthesised by heating **1** or **2** under acidic conditions.

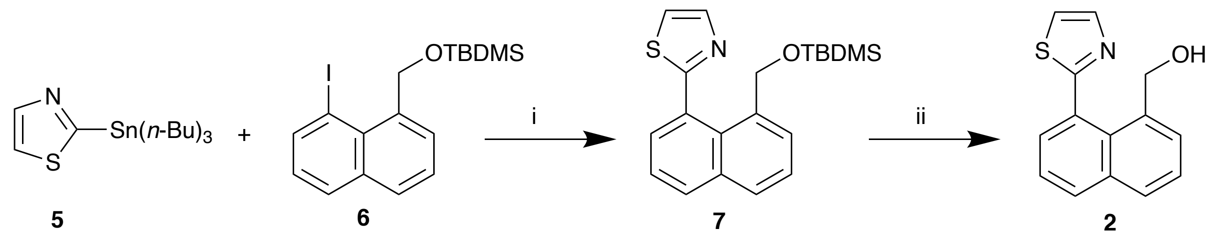

Scheme S1 Reactions and conditions: i) toluene, $Pd(PPh_3)_4$, Ar, 110 °C, 48 h; ii) $AcOH/THF/H_2O$, air, rt.

2-[8-({[Tert-butyldimethylsilyl]oxy)methyl}naphthalen-1-yl]thiazole **7**

A mixture of 2-(tri-*n*-butylstannyl)thiazole **5** (0.94 g, 2.5 mmol), *tert*-butyl[(8-iodonaphthalen-1-yl)methoxy]dimethylsilane **6**[2] (0.80 g, 2.0 mmol) and tetrakis(triphenylphosphine)palladium(0) (116 mg, 0.1 mmol) was dried under high vaccum for 30 min and then backfilled with argon. Then anhydrous toluene (20 mL) was added and the mixture was stirred under argon in an oil bath held at 110 °C for 48 h. After being allowed to cool to room temperature, the toluene was removed. Dichloromethane (50 mL) and water 50 (mL) were added and the organic layer separated. The organic phase was washed with aqueous potassium fluoride (5%, 2 × 20 mL), brine (50 mL), and water (50 mL), dried over anhydrous sodium sulfate, and filtered. The filtrate was collected and the solvent removed. The residue was purified by column chromatography over silica using dichloromethane as eluent to afford a slightly brownish solid of **7** (0.70 g, 98%). mp 73-74 °C. Elemental analysis (%) calcd for $C_{20}H_{25}NOSSi$ C 67.6, H 7.1, N 3.9, S 9.0; Found: C 67.3, H 7.25, N 3.9, S 8.8. λ_{max}(dichloromethane)/nm: 262 sh (logε/dm^3 mol^{-1} cm^{-1} 3.37), 297 (3.92). λ_{max}(fluorescence) (dichloromethane)/nm: 413. ^{1}H NMR (δ, 500 MHz, CD_2Cl_2): -0.05 (6 H, s, CH_3Si), 0.86 (9 H, s, CH_3), 4.40 (2 H, s, CH_2), 7.49 (1 H, dd, *J* = 7.0, *J* = 8.0, Naphthalenyl-H), 7.53 (1 H, dd, *J* = 1.5, *J* = 7.0, Naphthalenyl-H), 7.54 (1 H, d, *J* = 3.5, Thiazolyl-H), 7.56 (1 H, dd, *J* = 7.5, *J* = 7.5, Naphthalenyl-H), 7.85 (1 H, dd, *J* = 1.0, *J* = 8.0, Naphthalenyl-H), 7.91-7.93 (2 H, m, Naphthalenyl-H and Thiazolyl-H), 8.01 (1 H, dd, *J* = 1.5, *J* = 8.0, Naphthalenyl-H). ^{13}C NMR(δ, 125 MHz, CD_2Cl_2): -5.4,

18.6, 26.1, 64.1, 121.3, 124.2, 125.3, 126.4, 128.2, 129.2, 130.8, 131.2, 131.7, 134.9, 138.5, 142.8, 169.5. *m/z* (HRMS-ESI$^+$): expected for $[M+H]^+$ 356.1499, found 356.1501.

[8-(Thiazol-2-yl)naphthalen-1-yl]methanol **2**

A mixture of **7** (0.63 g, 1.8 mmol), glacial acetic acid (10 mL), tetrahydrofuran (4 mL) and water (4 mL) was stirred under air at room temperature for 24 h. Then saturated aqueous sodium hydrogen carbonate (250 mL) was added and the mixture extracted with dichloromethane (3 × 50 mL). The dichloromethane extracts were combined, dried over anhydrous sodium sulfate, and filtered. The filtrate was collected and the solvent removed. The residue was purified by column chromatography over silica using dichloromethane:ethyl acetate (10:0-10:3) as eluent to give a white solid of **2** (0.28 g, 66%). mp 105-106 °C; mp(DSC) = 107 °C (onset), 111 °C (peak) (first heating scan - scan rate 100 °C/min). Tg = 8 °C (second heating scan - DSC scan rate 100 °C/min). $T_{5\%}$ = 244 °C. Elemental analysis (%) calcd for $C_{14}H_{11}NOS$ C 69.7, H 4.6, N 5.8, S 13.3; Found: C 69.4, H 4.8, N 5.9, S 13.4. IR ν_{max}/cm^{-1} 3226 (OH). λ_{max}(dichloromethane)/nm: 298 (logε/dm^3 mol^{-1} cm^{-1} 3.97). λ_{max}(fluorescence) (dichloromethane)/nm: 419. ^{1}H NMR (δ, 500 MHz, CD_2Cl_2): 4.18 (3 H, brs, OH and CH_2), 7.50-7.57 (3 H, m, Naphthalenyl-H), 7.60 (1 H, d, *J* = 3.5, Thiazolyl-H), 7.61 (1 H, dd, *J* = 1.5, *J* = 7.0, Naphthalenyl-H), 7.88 (1 H, d, *J* = 3.5, Thiazolyl-H), 7.91 (1 H, dd, *J* = 1.5, *J* = 8.0, Naphthalenyl-H), 8.03 (1 H, dd, *J* = 1.5, *J* = 8.0, Naphthalenyl-H). ^{13}C NMR(δ, 125 MHz, CD_2Cl_2): 64.1, 121.7, 124.5, 126.7, 129.7, 130.1, 130.2, 131.3, 132.2, 132.3, 135.6, 138.3, 142.7, 170.9. *m/z* (HRMS-ESI$^+$): expected for $[M-OH]^+$ 224.0528, found 224.0527; expected for $[M+Na]^+$ 264.0454, found 264.0453.

7H-Benzo[de]pyrido[2,1-a]isoquinolin-8-ium bromide **1’**

A mixture of **1**[1] (0.13 g, 0.53 mmol), glacial acetic acid (8 mL) and hydrobromic acid (48%, 8 mL) was stirred at reflux under argon in an oil bath for 6 h. After being allowed to cool to room temperature, water (100 mL) was added and the mixture was extracted with dichloromethane (4 × 50 mL) and a mixture of dichloromethane:methanol (4:1) (2 × 50 mL). The organic extracts were combined, dried over anhydrous sodium sulfate, and filtered. The filtrate was collected and the solvent removed. The residue was purified by recrystallization with methanol/diethyl ether three times to give **1’** as a yellow solid (93 mg, 59%). mp 229-230 °C. Elemental analysis (%) calcd for $C_{14}H_{11}NBr$ C 64.45, H 4.1, N 4.7; Found: C 64.1, H 4.1, N 4.4. λ_{max}(ethanol)/nm: 276 (logε/dm^3 mol^{-1} cm^{-1} 3.95), 327 sh (3.48), 346 sh (3.62), 376 (3.86). λ_{max}(fluorescence, λ_{ex} = 378 nm) (ethanol)/nm: 479. ^{1}H NMR (δ, 500 MHz, CD_3OD): 6.28 (2 H, s, CH_2), 7.67-7.69 (1 H, m, Naphthalenyl-H), 7.72 (1 H, dd, *J* = 7.0, *J* = 7.0, Naphthalenyl-H), 7.79 (1 H, dd, *J* = 8.0, *J* = 8.0, Naphthalenyl-H), 7.98 (1 H, ddd, *J* =

1.5, J = 6.0, J = 7.5, Pyridyl-H), 8.01 (1 H, dd, J = 1.0, J = 8.0, Naphthalenyl-H), 8.25 (1 H, d, J = 8.5, Naphthalenyl-H), 8.58 (1 H, ddd, J = 1.5, J = 7.0, J = 8.5, Pyridyl-H), 8.62 (1 H, d, J = 7.5, Naphthalenyl-H), 8.89 (1 H, ddd, J = 1.0, J = 1.0, J = 6.5, Pyridyl-H), 8.93 (1 H, d, J = 8.0, Pyridyl-H). ^{13}C NMR(δ, 125 MHz, CD_3OD): 58.2, 123.1, 125.3(8), 125.3(9), 126.2, 126.3, 126.8, 127.1, 128.1, 128.8(1), 128.8(3), 134.2, 135.3, 146.2, 146.6, 150.3. *m*/*z* (HRMS-ESI$^+$): expected for [M-Br]$^+$ 218.0964, found 218.0963.

7H-Benzo[de]thiazolo[2,3-a]isoquinolin-8-ium bromide **2'**

A mixture of **2** (0.10 g, 0.42 mmol), glacial acetic acid (8 mL) and hydrobromic acid (48%, 8 mL) was stirred at reflux under argon in an oil bath for 6 h. After being allowed to cool to room temperature, the acids were removed under vacuum. The residue was dissolved in acetonitrile (2 mL) and then diethyl ether (10 mL) was added slowly. The precipitate was collected and dried under vacuum to give **2'** as a yellow solid (74 mg, 58%). λ_{max}(ethanol)/nm: 267 (logε/dm^3 mol^{-1} cm^{-1} 3.93), 282 sh (3.71), 327 sh (3.33), 344 sh (3.59), 377 (3.90). λ_{max}(fluorescence, λ_{ex} = 377 nm) (ethanol)/nm: 466. ^{1}H NMR (δ, 500 MHz, DMSO-d_6): 6.18 (2 H, s, CH_2), 7.71-7.76 (3 H, m, Naphthalenyl-H), 8.04 (1 H, dd, J = 1.0, J = 7.5, Naphthalenyl-H), 8.30 (1 H, d, J = 8.0, Naphthalenyl-H), 8.36 (1 H, d, J = 4.0, Thiazoyl-H), 8.37 (1 H, dd, J = 0.5, J = 7.0, Naphthalenyl-H), 8.55 (1 H, d, J = 4.0, Thiazoyl-H). ^{13}C NMR(δ, 125 MHz, DMSO-d6): 52.8, 119.6, 123.20, 123.22, 125.2, 126.0, 126.1, 126.8, 127.1, 127.9, 132.1, 134.5, 137.0, 164.8. *m*/*z* (HRMS-ESI$^+$): expected for [M-Br]$^+$ 224.0528, found 224.0524.

2-[8-(Hydroxymethyl)naphthalen-1-yl]pyridin-1-ium chloride **1-H$^+$ Cl$^-$**

Hydrogen chloride (generated *in situ* from adding hydrochloric acid (37%) dropwise to calcium chloride[3]) was bubbled through a solution of **1** (50 mg, 0.21 mmol) in anhydrous diethyl ether (20 mL) stirred at room temperature under argon. The precipate formed after 10 min was collected at the filter under nitrogen flow, washed with anhydrous diethyl ether (20 mL) and then dried under vacuum. The residue was recrystallized from anhydrous acetonitrile and diethyl ether to give **1-H$^+$ Cl$^-$** as a white solid (48 mg, 86%). λ_{max}(acetonitrile)/nm: 261 (logε/dm^3 mol^{-1} cm^{-1} 4.02), 287 (3.82), 292 sh (3.81), 328 sh (3.58). λ_{max}(fluorescence, λ_{ex} = 335 nm) (acetonitrile)/nm: 482. ^{1}H NMR (δ, 500 MHz, DMSO-d_6): 4.09 (2 H, s, CH_2), 7.58 (1 H, dd, J = 1.0, J = 7.0, Naphthalenyl-H), 7.61-7.67 (2 H, m, Naphthalenyl-H), 7.76 (1 H, dd, J = 1.0, J = 7.0, Naphthalenyl-H), 8.03-8.07 (3 H, m, Naphthalenyl-H and Pyridinium-H), 8.22 (1 H, dd, J = 1.5, J = 8.0, Naphthalenyl-H), 8.54 (1 H, ddd, J = 1.5, J = 8.0, J = 8.0, Pyridinium-H), 8.89 (1 H, m, Pyridinium-H). ^{13}C NMR (δ, 125 MHz, DMSO-d6): 62.6, 124.3, 125.3, 126.2, 127.6, 128.0, 128.1, 128.7, 130.3, 131.1, 132.0, 134.3, 137.6, 142.1, 143.9, 155.6. *m*/*z* (HRMS-ESI$^+$): expected for [M-Cl]$^+$ 236.1070, found 236.1071.

2-(Naphthalen-1-yl)pyridin-1-ium chloride **3-H⁺ Cl⁻**

Hydrogen chloride (generated *in situ* from adding hydrochloric acid (37%) dropwise to calcium chloride[3]) was bubbled through a solution of **3** (205 mg, 1.0 mmol) in anhydrous diethyl ether (10 mL) stirred at room temperature under argon. The precipate formed after 10 min was collected at the filter under nitrogen flow, washed with anhydrous diethyl ether (20 mL) and then dried under vacuum. The residue was recrystallized from anhydrous acetonitrile and diethyl ether to give **$3\text{-}H^+$ Cl^-** as a white solid (210 mg, 87%). λ_{max}(acetonitrile)/nm: 256 (logε/dm^3 mol^{-1} cm^{-1} 4.01), 283 sh (3.70), 294 sh (3.72), 311 sh (3.75), 318 sh (3.76), 325 (3.77). λ_{max}(fluorescence, λ_{ex} = 335 nm) (acetonitrile)/nm: 471. ^{1}H NMR (δ, 500 MHz, CD_3OD): 7.63-7.69 (2 H, m, Naphthalenyl-H), 7.71-7.75 (1 H, m, Naphthalenyl-H), 7.76-7.80 (2 H, m, Naphthalenyl-H), 8.07-8.12 (2 H, m, Naphthalenyl-H), 8.18-8.21 (1 H, m, Pyridinium-H), 8.22 (1 H, brm, Naphthalenyl-H), 8.31-8.33 (1 H, m, Pyridinium-H), 8.79 (1 H, ddd, *J* = 1.5, *J* = 8.0, *J* = 8.0, Pyridinium-H), 8.96-8.98 (1 H, m, Pyridinium-H). ^{13}C NMR(δ, 125 MHz, CD_3OD): 124.7, 126.4, 127.4, 128.3, 129.5, 130.1, 130.3, 130.4, 130.7, 131.6, 133.5,135.3, 143.4, 148.3, 154.0. *m/z* (HRMS-ESI$^+$): expected for $[M\text{-}Cl]^+$ 206.0964, found 206.0960.

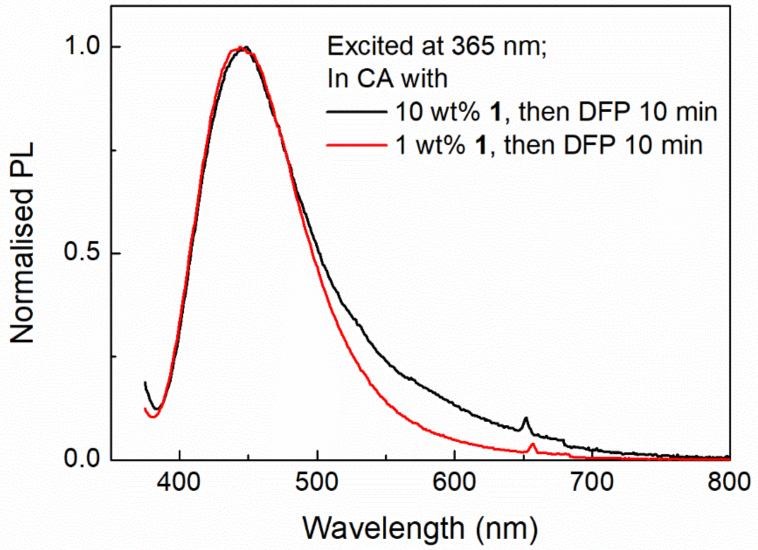

Supplementary Figure S1. Comparison of PL spectra of cellulose acetate films with 1 wt% or 10 wt% **1** after exposing to aged DFP. PL spectra were recorded on a FS5 spectrometer at excitation wavelength of 365 nm.

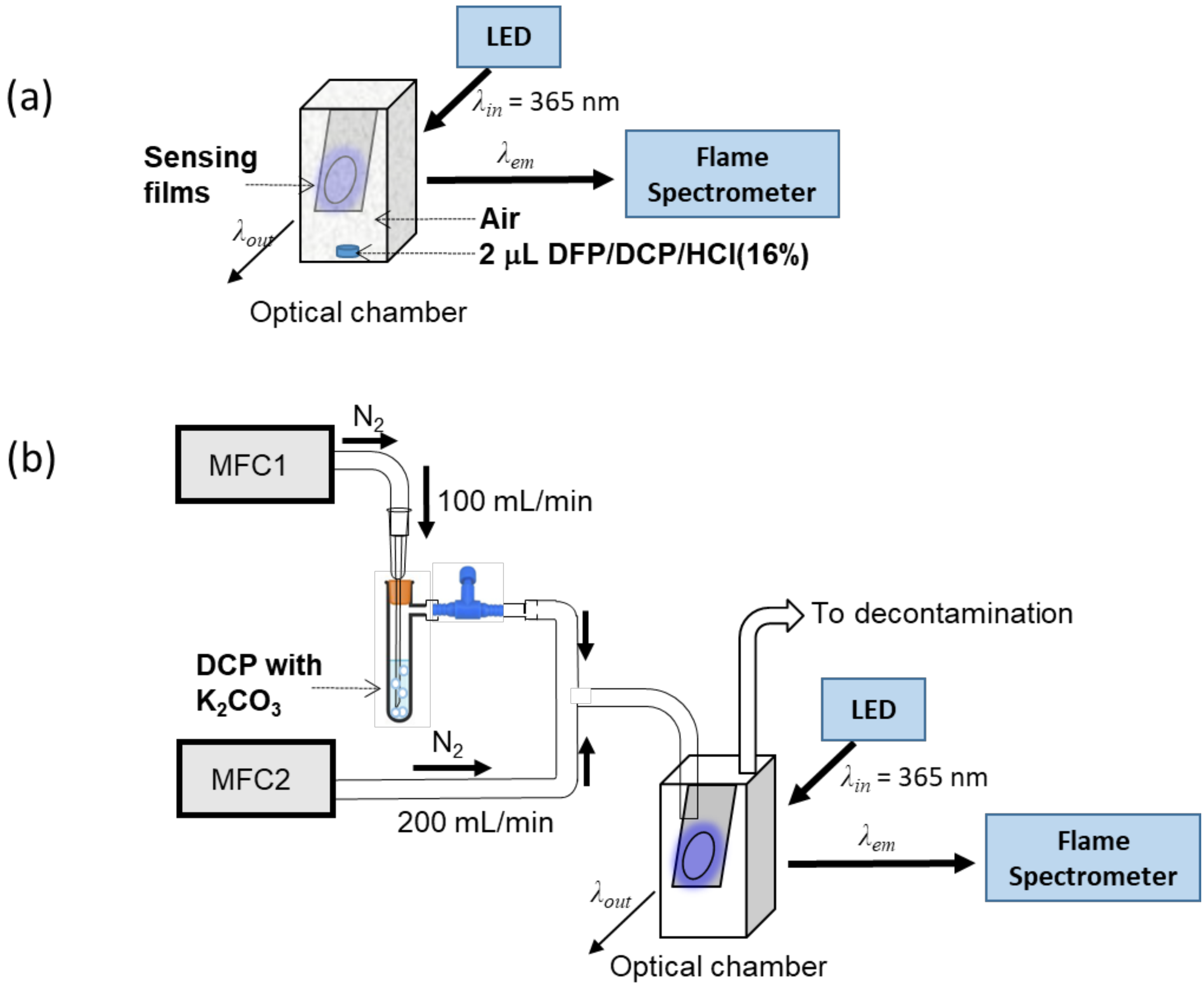

Supplementary Figure S2. Experimental setup for the vapor detection by the sensing films under (a) ambient conditions and (b) nitrogen. For setup (a), 2 µL of DCP or DFP or HCl (aq. 16%) in a Teflon lid was placed at the bottom of a titanium optical chamber (volume: 20 mL) to allow vaporization to generate the analyte vapor. In setup (b), DCP vapor was generated by bubbling nitrogen at 100 mL/min through liquid DCP liquid (1 mL in a 10 mL Schlenk tube, with or without 200 mg of potassium carbonate), which was then diluted by a second nitrogen flow (200 mL/min) and introduced to the titanium optical chamber.

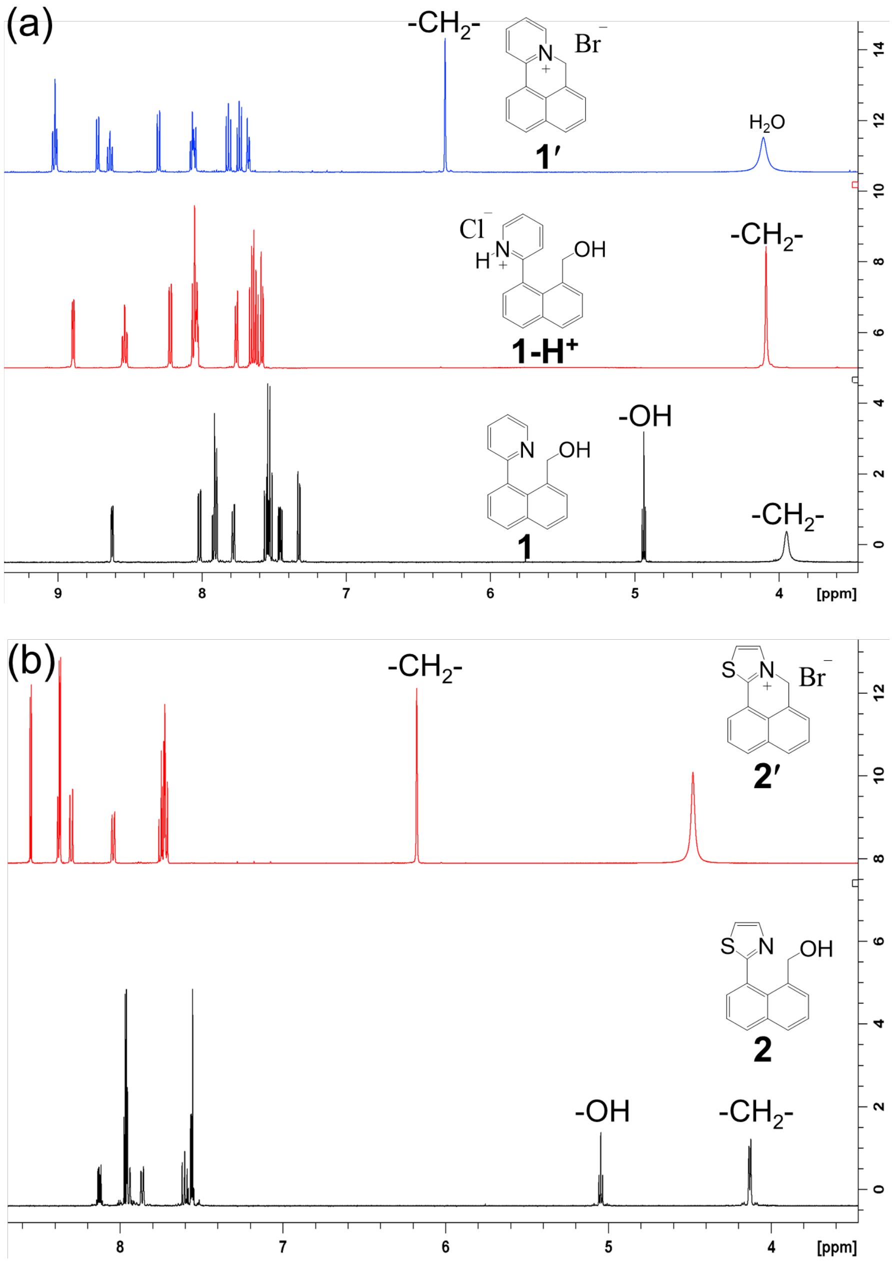
(a)
-CH2-
Br
N
1′
H2O
Cl
H
N
OH
-CH2-
1-H+
-OH
N
OH
1
-CH2-
9
8
7
6
5
4
[ppm]
14
12
10
8
6
4
2
0
(b)
-CH2-
S
N
Br
2′
S
N
OH
2
-OH
-CH2-
8
7
6
5
4
[ppm]
12
10
8
6
4
2
0

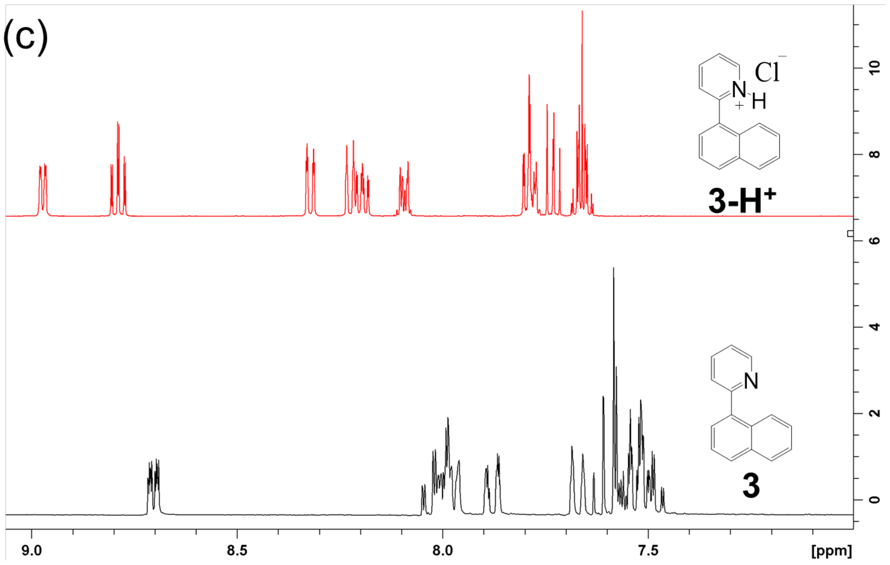

Supplementary Figure S3. ^{1}H NMR spectra of (a) **1**, **1-H**$^+$, **1'**, (b) **2** and **2'** in DMSO-d6, (c) and **3**, **3-H**$^+$ in CD_3OD. The downfield shifts of the proton alpha to the nitrogen atom is consistent with moving from a neutral to a charged species.

(a)

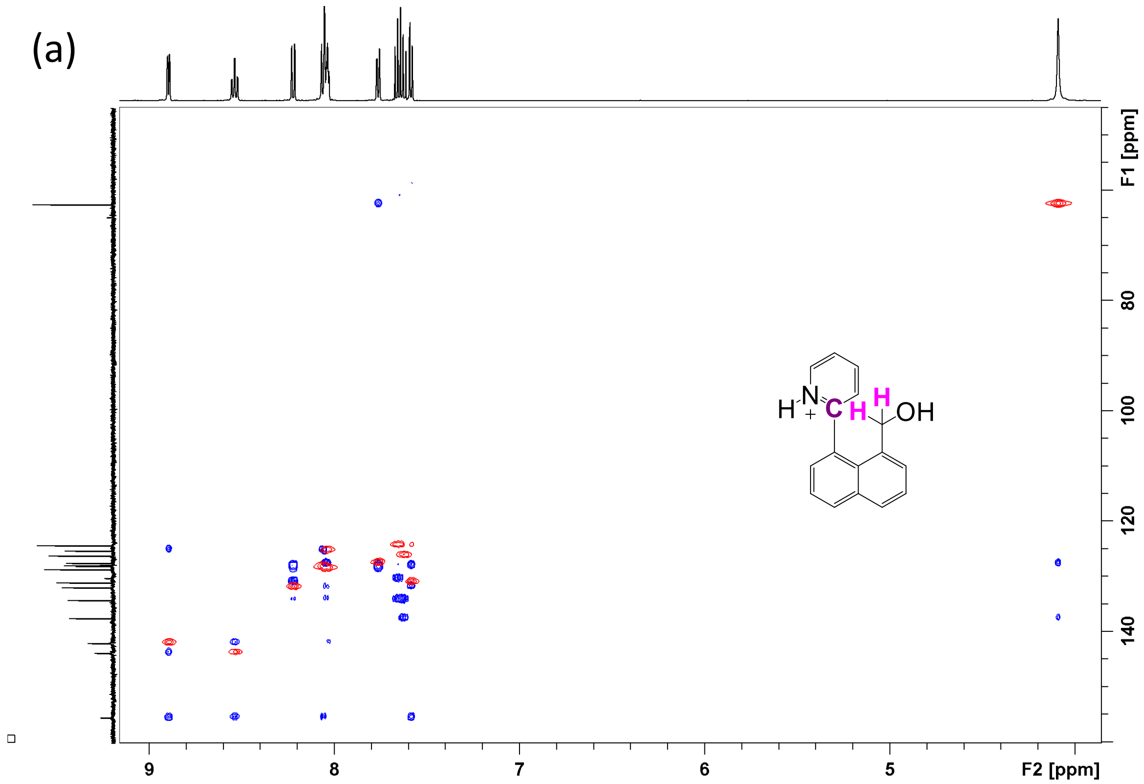
F1 [ppm]
80
100
120
140
9
8
7
6
5
F2 [ppm]
H
N
+
C
H
H
OH

(b)

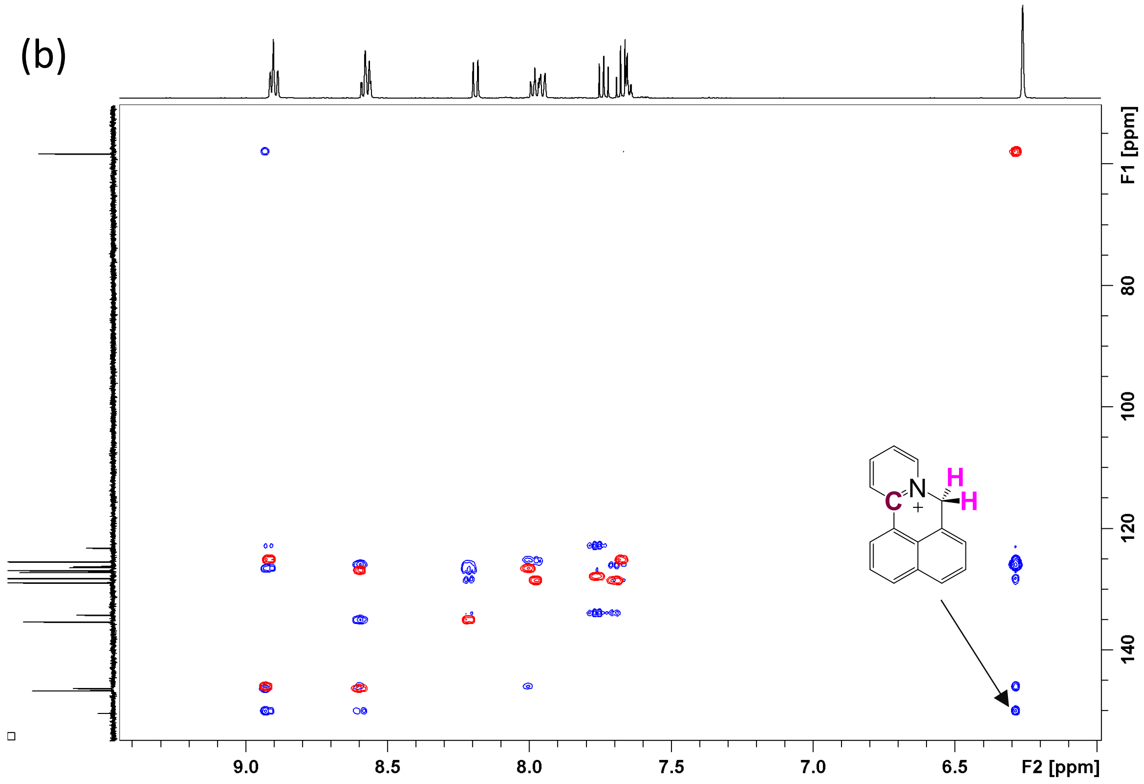
F1 [ppm]
80
100
120
140
9.0
8.5
8.0
7.5
7.0
6.5
F2 [ppm]
C
N
+
H
H

(c)

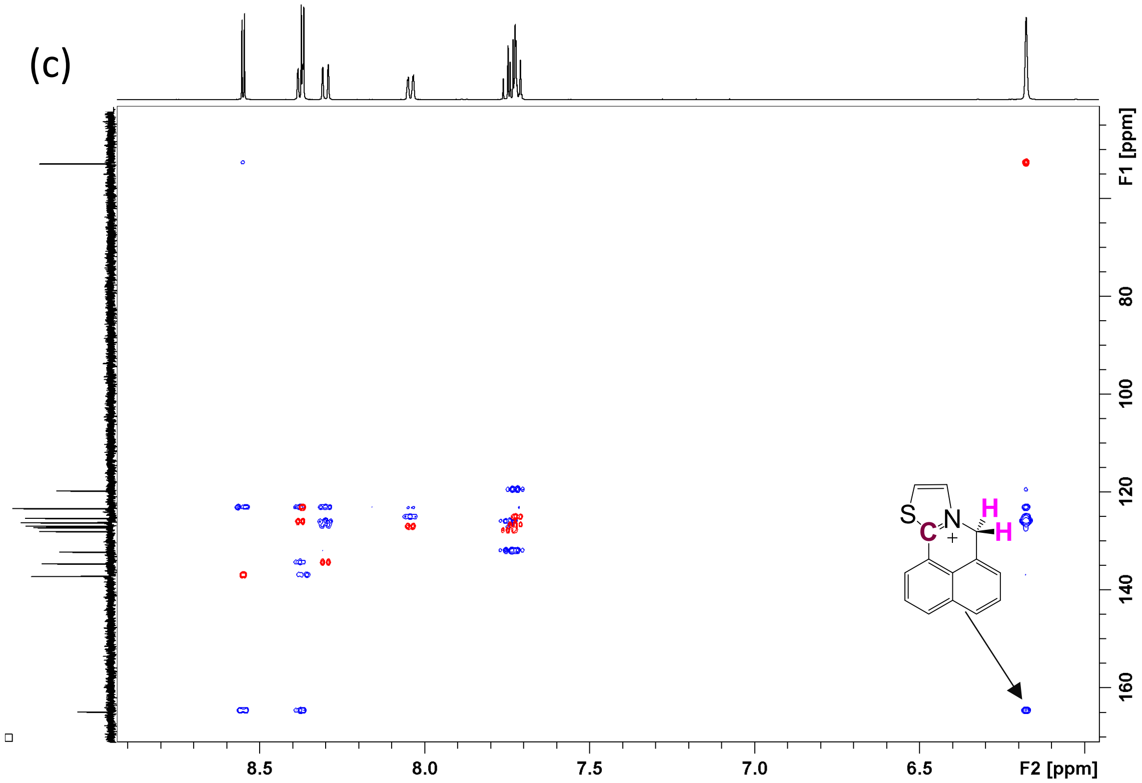

Supplementary Figure S4. HMBC 2D NMR of (a) **1-H**$^{+}$ in DMSO-d$_6$, (b) **1’** in CD_3OD, (c) and **2’** in DMSO-d$_6$. The cyclised compounds **1’** and **2’** show correlations between the CH_2 protons and the carbon alpha to the nitrogen while **1-H**$^{+}$ does not.

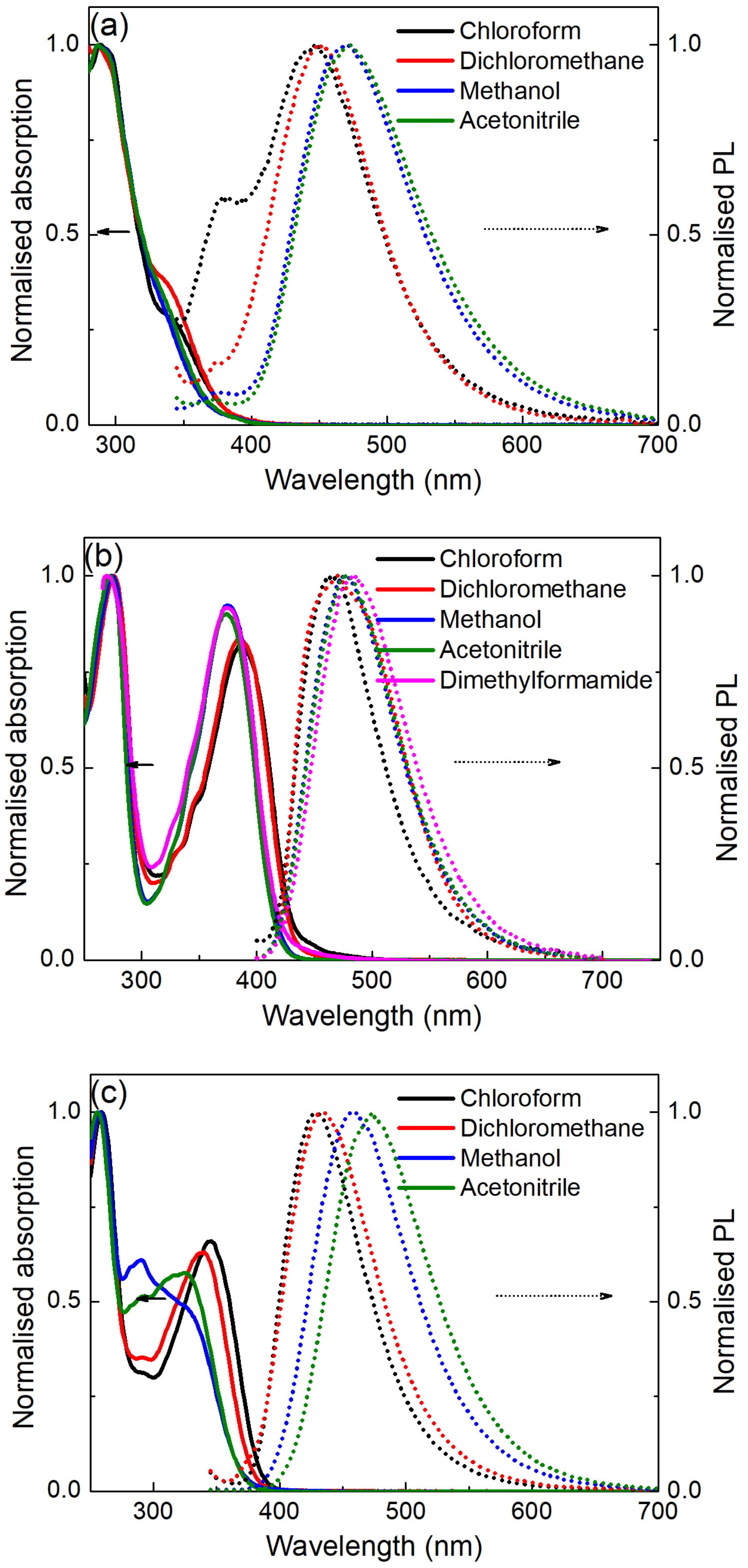

Supplementary Figure S5. UV-vis and PL spectra of (a) **1-H**$^+$, (b) **1'**, (c) and **3-H**$^+$ in different solvents illustrating the solvatochromic effect. The dielectric constants are 4.8, 8.9, 32.7, 37.5 and 36.7 for chloroform, dichloromethane, methanol, acetonitrile and dimethylformamide, respectively. The three compounds have low solubility in 1,4-dioxane, ethyl acetate and tetrahydrofuran. The excitation wavelength was 335 nm for **1-H**$^+$ and **3-H**$^+$ and 377 nm for **1'**.

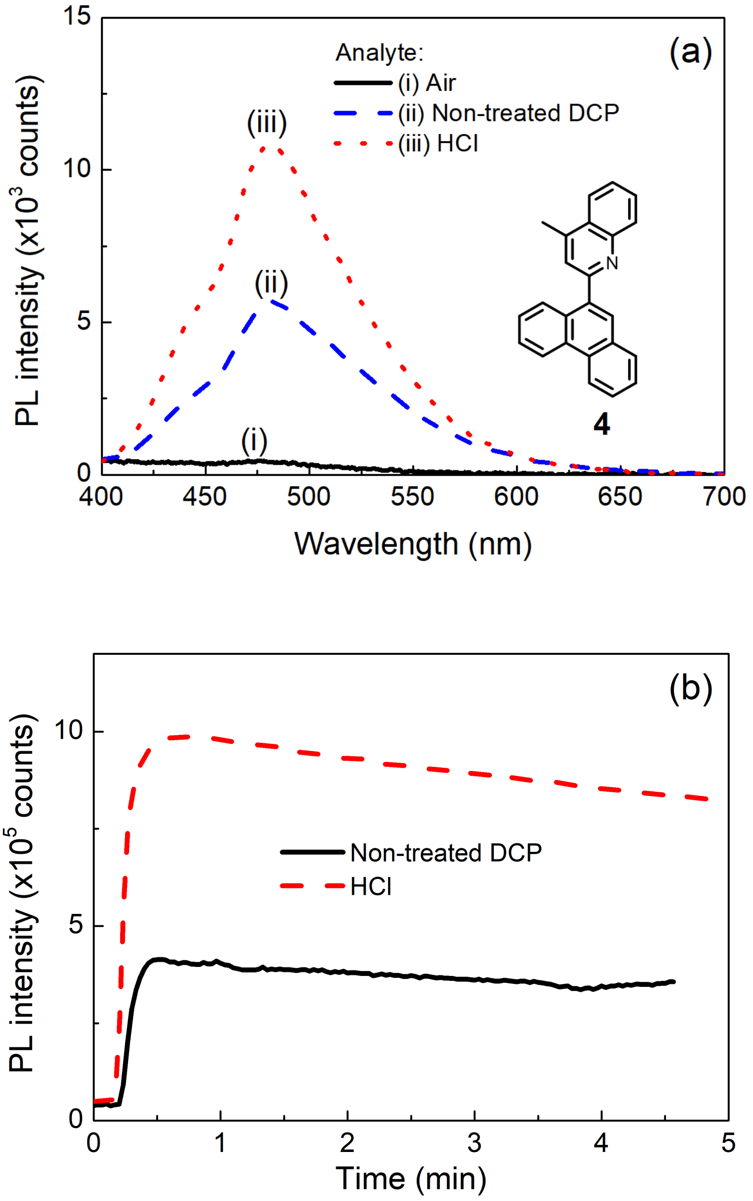

Supplementary Figure S6. (a) PL spectra (b) and time-dependent PL intensity of 10 wt% **4**:cellulose acetate films before and after continuous exposure to non-treated DCP and HCl using the setup described in Supplementary Figure S2a. Hydrogen chloride vapor was generated from the natural vaporisation of 2 μL of 16% aqueous hydrochloric acid.

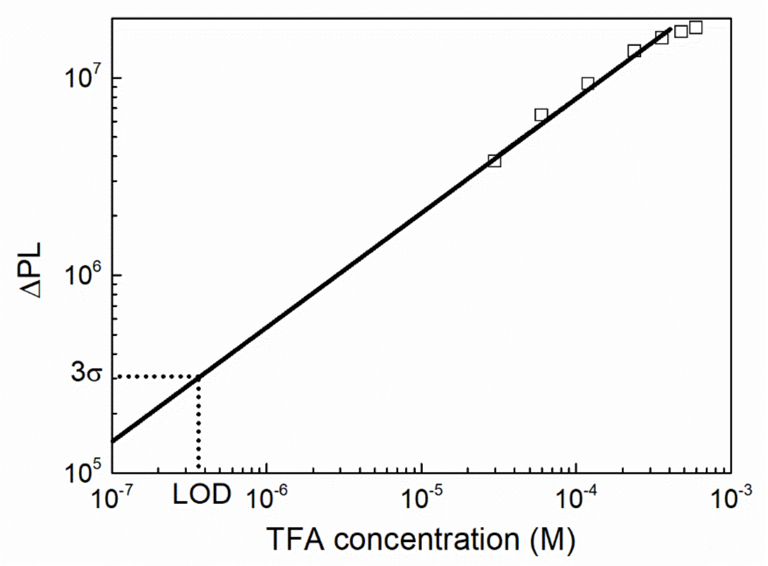

Supplementary Figure S7. ΔPL of **1** in dichloromethane (3×10^{-5} M) upon addition of TFA (1-20 equiv). ΔPL is the change of PL intensity at 463 nm. The excitation wavelength is 335 nm. 3σ is three times standard deviation. LOD is limit of detection, which by extrapolation is ≈$4x10^{-7}$ M.

Supplementary references

1. Zhang, S.-W. & Swager, T. M. Fluorescent detection of chemical warfare agents: functional group specific ratiometric chemosensors. *J. Am. Chem. Soc.* **125**, 3420–3421 (2003).
2. Anderson, B. J. *et al.* Platinum-catalyzed enantioselective tandem alkylation/arylation of primary phosphines. Asymmetric synthesis of P-stereogenic 1-phosphaacenaphthenes. *Org. Lett.* **10**, 4425–4428 (2008).
3. Francisco, J. A. A convenient way to generate hydrogen chloride in the Freshman lab. *J. Chem. Educ.* **72**, 1139 (1995).
